# Lactylation stabilizes PD-L1 to promote tumor immune evasion and cell growth

**DOI:** 10.1038/s41419-026-08589-1

**Published:** 2026-03-21

**Authors:** Lijun Liang, Yiqi Zong, Jinghao Huang, Yixuan Chen, Nuotong Xu, Pengyu Yan, Hai Song, Ming Wu

**Affiliations:** 1 Department of Thoracic Surgery, Second Affiliated Hospital, School of Medicine, Zhejiang University, Hangzhou, China; 2 Zhejiang University School of Medicine, Hangzhou, China; 3 Department of Respiratory and Critical Care Medicine, Center for Oncology Medicine, the Fourth Affiliated Hospital of School of Medicine, and International School of Medicine, International Institutes of Medicine, Zhejiang University, Yiwu, China

**Keywords:** Lung cancer, Post-translational modifications

## Abstract

Programmed death-ligand 1 (PD-L1) plays a critical role in tumor immune evasion, yet the mechanisms that regulate its expression, specifically the metabolic control of its stability and function, remain elusive. In this study, we demonstrate that lactate, a key metabolite in the tumor microenvironment, upregulates PD-L1 expression via lysine lactylation (Kla) of PD-L1 at residue K280 within its intracellular domain. This modification stabilizes PD-L1 by inhibiting E3 ligase HUWE1 binding, ubiquitination, and subsequent proteasomal degradation. We identified alanyl-tRNA synthetase 1 (AARS1) as the lactyltransferase that utilizes lactate as a lactyl-donor and is responsible for PD-L1 K280 lactylation. Functionally, PD-L1 lactylation promotes tumor immune evasion by impairing CD8 + T cell-mediated cytotoxicity and accelerates tumor growth in vivo. Furthermore, sodium lactate (NaLa) administration enhances the efficacy of anti-PD-L1 immunotherapy in preclinical models. Clinically, PD-L1 K280 lactylation correlates with advanced non-small cell lung cancer stages and poor patient survival, highlighting its potential as a diagnostic biomarker. Our findings unveil a novel lactate-PD-L1 regulatory axis and propose lactylation as a therapeutic target to augment the efficacy of the immune checkpoint blockade.

## Introduction

Programmed death-ligand 1 (PD-L1) is an immune checkpoint protein on tumor cells, and its engagement with programmed cell death protein-1 (PD-1) on T cells leads to suppression of the tumor-killing activity of cytotoxic T lymphocytes, allowing cancer cells to evade the immune system [1]. PD-L1 and PD-1 inhibitors have produced remarkable clinical outcomes and become a standard of care in the treatment of many types of cancer, including non-small cell lung cancer (NSCLC), breast cancer, and melanoma. However, for several cancer types, the response rate is approximately 20%, and only a small subset of patients achieve durable responses [1]. Importantly, it has been suggested that the high expression of PD-L1 in tumors is a biomarker indicating improved sensitivity to PD-L1 or PD-1 blockades [2, 3]. Therefore, discovering the mechanism modulating PD-L1 may lead to the development of mechanism-driven treatment strategies that can improve cancer response rates and patient survival.

In 2019, Zhang et al. [4] pioneered the identification and characterization of histone lactylation, a post-translational modification (PTM) driven by lactate, using peptide immunoprecipitation coupled with high-sensitivity HPLC-MS/MS analysis. Since then, lactate and lactylation have offered new avenues for understanding the intricate interplay between cell metabolism and gene expression through epigenetic regulation. Subsequent research has revealed that lactylation occurs in both histone and non-histone proteins, which exert diverse functions in diverse cellular physiological and pathological processes. PD-L1 expression is tightly regulated at transcriptional and post-translational levels [5]. Various inflammatory cytokines, such as interferon-γ secreted by immune cells, are the major inducers that could transcriptionally activate PD-L1 expression [5, 6]. However, diverse PTMs including glycosylation, acetylation, phosphorylation, palmitoylation, and ubiquitination could modulate the expression and other functional implications of PD-L1 [5, 7–11]. However, whether novel PTM lactylation affects PD-L1 remains underexplored.

In this study, we report that PD-L1 could be lactylated at K280, and lactylation-stabilized PD-L1 may occur by antagonizing its polyubiquitination. We further demonstrate that alanyl-tRNA synthetase 1 (AARS1) is the lactyltransferase that drives lactylation, and establish its functional significance in immune evasion and tumor progression. Clinically, PD-L1 lactylation correlates with non-small cell lung cancer (NSCLC) aggressiveness, underscoring its translational relevance. Our study elucidates a metabolic-immune crosstalk wherein lactate fuels PD-L1-mediated immune suppression, offering new strategies for potentiating cancer immunotherapy.

## Results

### PD-L1 could be modulated by Lactate at transcriptional or translational levels

**Fig. 1:**
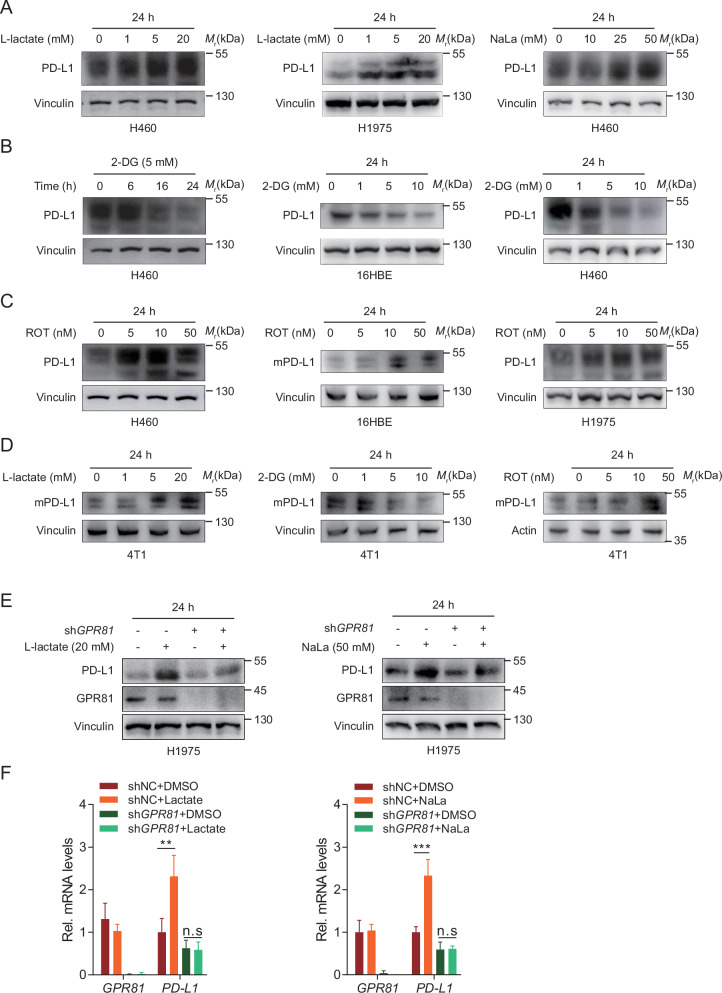
Lactate increased PD-L1 expression at the protein and mRNA levels. **A** Western blot indicated increased PD-L1 expression following lactate or NaLa treatment, as indicated for 24 h in H460 and H1975 cells. *Mr*, relative molecular mass. **B** Western blot demonstrated decreased PD-L1 expression with 2-deoxy-d-glucose (2-DG) treatment as indicated in H460 and 16HBE cells. **C** Western blot displayed increased PD-L1 expression following treatment with different concentrations of rotenone (ROT) for 24 h. **D** 4T1 cells were exposed different concentrations of lactate, 2-DG, and ROT as indicated and harvested at 24 h and the expression of mPD-L1 was detected. **E**, **F** H1975 cells that stably expressed GPR81 shRNA were treated with lactate 20 mM or NaLa 50 mM for 24 h, lysed, and cell lysates were analyzed using western blot (**E**) and quantitative real-time RT–PCR (**F**). Two-sided unpaired Student’s t-test; **P < 0.01, ***P < 0.001; n.s. not significant.

Given the role of lactate-enriched microenvironment in the creation of a niche that favors tumor growth, we hypothesized that lactate may regulate the PD-1/PD-L1 pathway, thereby conferring advantages over anti-tumor immune surveillance. Treatment of NSCLC cell lines (H460, H1975) with sodium lactate (NaLa) or lactate (the cell culture medium was calibrated to pH 7.4) induced a significant dose- and time-dependent upregulation of the PD-L1 protein (Fig. 1A). We next examined the effect of a glycolysis inhibitor, 2-deoxy-d-glucose (2-DG), on PD-L1 expression and found that 2-DG decreased PD-L1 in a dose- and time-dependent manner in H460 and human bronchial epithelial cell line 16HBE cells (Fig. 1B). In contrast, rotenone (ROT), an inhibitor of the mitochondrial respiratory chain that drives cells toward glycolysis for energy supply—triggered a dose-dependent increase in PD-L1 expression in H460, H1975, and 16HBE cells (Fig. 1C). To ensure our study is universal, we examined the influence of lactate and the two compounds on mouse PD-L1 (mPD-L1) expression in a different histological mice breast cancer line, 4T1. Consistently, western blot analysis indicated that lactate and ROT induced PD-L1 upregulation, and 2-DG significantly decreased mPD-L1 expression in 4T1 cells (Fig. 1D). These data suggested that glycolytic product lactate can upregulate PD-L1 expression among different cell lines.

It has been reported that the G protein-coupled receptor 81 (GPR81), found on the cell membrane, mediates lactate induction of PD-L1 transcriptional expression through the activation of the transcriptional coactivator TAZ, and furthermore PD-L1 promoter activity enhancement [12]. We found that the PD-L1 mRNA levels decreased in parallel with protein levels after GPR81 knockdown in H1975 cells (Fig. 1E, F). Lactate or NaLa treatment significantly upregulated PD-L1 expression at the protein level in GPR81 silenced H1975 cells; however, this induction was not observed at the mRNA level (Fig. 1E, F). These results indicated that lactate promoted PD-L1 expression through GPR81-dependent and -independent pathways.

### PD-L1 could be modified by lysine lactylation (Kla)

It has been reported that Kla modification can occur on non-histone proteins across various normal or cancer cells. Therefore, we went on to confirm Kla modification in NSCLC cells. Figure S1 shows that multiple protein bands with different molecular weights were detected at the whole line. This result indicated that the vast majority of non-histone proteins were lactylated in NSCLC cells. As expected, lactate or NaLa increased the abundance of Kla in H460, H1299, and Lewis lung cancer (LLC, a mouse lung cancer cell line), whereas these results were not observed in the NaCl administration group (Fig. S1A). Furthermore, 2-DG decreased while ROT increased the whole Kla levels in a dose- and time-dependent manner (Fig. S1B, C). Together, these observations demonstrated that Kla modified non-histone proteins that widely exist in NSCLC cells, and they were determined by extracellular lactate (NaLa) and endogenous produced lactate.

**Fig. 2:**
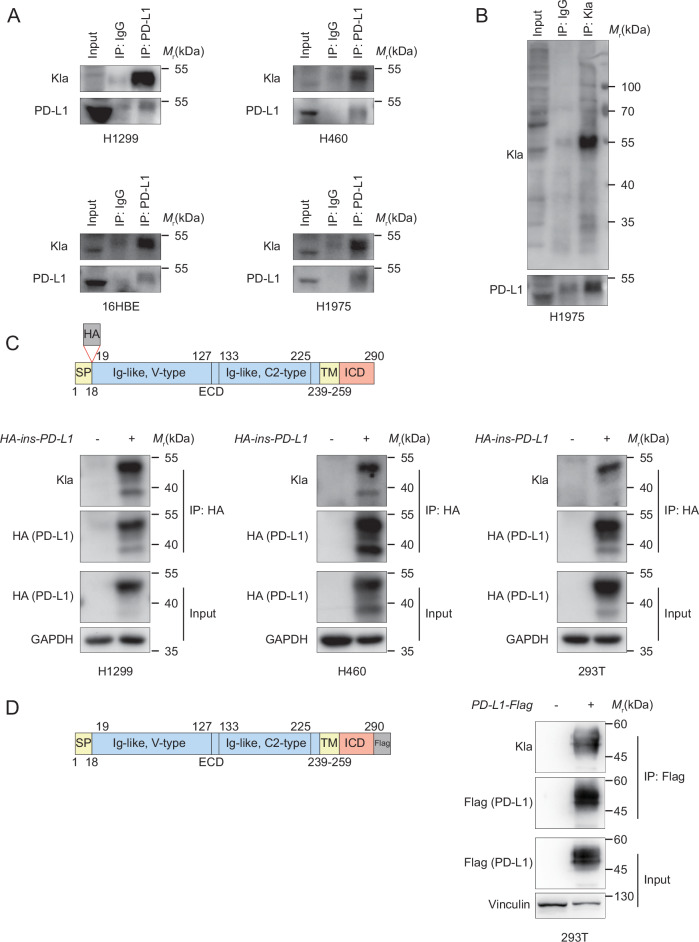
PD-L1 could be lactylated. **A** Immunoblot (IB) analysis of the anti-PD-L1 immunoprecipitates (IPs) derived from the different cell lines. IgG was used as a negative control. **B** IB analysis of the anti-Kla IPs derived from the H1975 cells. **C** Schematic of the N-terminal HA-tag-inserted PD-L1 (HA-ins-PD-L1) plasmid. SP signal peptide, ECD extracellular domain, TM transmembrane domain, ICD intracellular domain (upper). IB analysis of the anti-HA IPs derived from the different cells transfected with HA-ins-PD-L1 (bottom). **D** Schematic of the C-terminal Flag-tag PD-L1 (PD-L1-Flag) plasmid. (left). IB analysis of the anti-Flag IPs derived from the 293 T cells transfected with PD-L1-Flag (right).

Given that lactate or NaLa could modulate PD-L1 expression through a non-transcriptional pathway and considering the presence of amounts of lactylated non-histone proteins in NSCLC cells, we wondered whether PD-L1 could be directly modified by Kla. To this end, we first observed that lactylation of endogenous PD-L1 was detected by a pan-lactyl lysine antibody in multiple NSCLC cell lines and 16HBE cells (Fig. 2A). Furthermore, PD-L1 was found in the pan-lactyl lysine antibody immunoprecipitated Kla modified proteins in H1975 cells (Fig. 2B). The signal peptide would be cleaved during PD-L1 protein maturation and migration to the cell membrane. We therefore generated a N-terminal HA-tag-inserted PD-L1 (HA-ins-PD-L1) plasmid that had HA sequences inserted after the signal peptide sequence within the full-length of PD-L1 (Fig. 2C). As shown in Fig. 2C, cells were transfected with HA-ins-PD-L1, and Kla modified PD-L1 was detected using the pan-lactyl lysine antibody following immunoprecipitation with α-HA beads. The plasmid Flag tag fused to the N-terminal of PD-L1 was also utilized and transfected to 293 T. Lysine lactylated PD-L1 was also strongly detected in the cellular lysates after being immunoprecipitated with anti-Flag beads (Fig. 2D). Collectively, these results unveiled a novel post-translational modification of PD-L1.

### PD-L1 is lactylated at Lys 280 within its intracellular domain

**Fig. 3:**
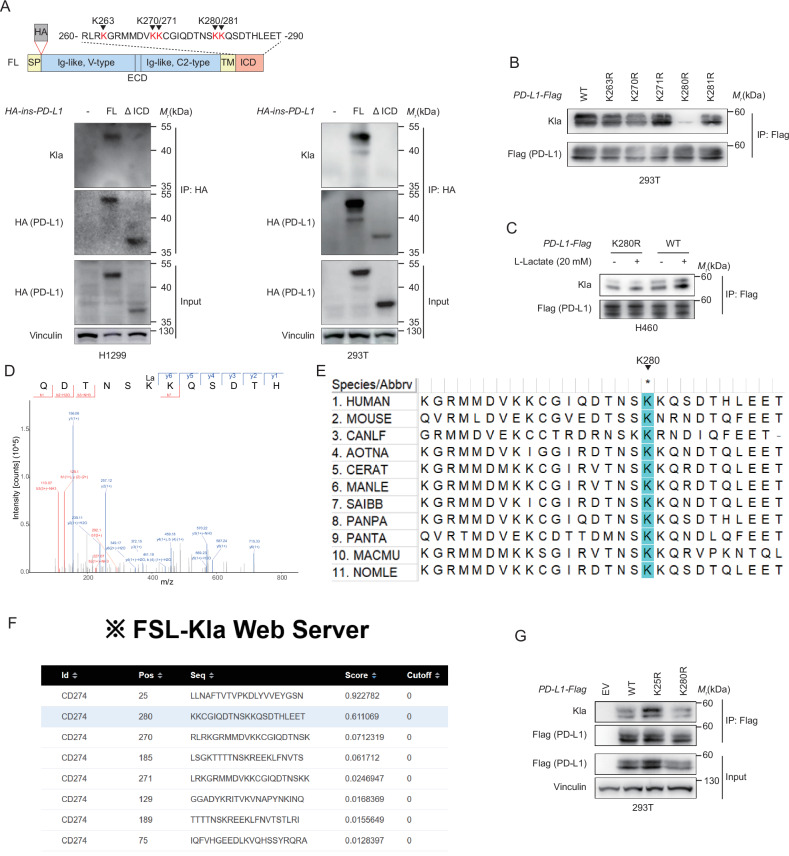
Lysine 280 as the lactylation modification site of PD-L1. **A** Schematic of the HA-ins-PD-L1 protein domains and amino acid residues in the ICD (upper). IB analysis of the anti-HA IPs derived from the H1299 and 293 T cells transfected with the HA-ins-PD-L1 (wild type [WT]) or the deletion mutant of the ICD (amino acids 260–290) (bottom). **B** IB analysis of the anti-Flag IPs derived from the 293 T cells transfected with the PD-L1-Flag WT or a series lysine (K) to arginine (R) mutants. **C** IB analysis of the anti-Flag IPs derived from the H460 cells transfected with the PD-L1-Flag WT or K280R and treated with lactate 20 mM for 24 h. **D** The MS/MS spectra of the lactylated PD-L1 peptide for the identification and quantification of K280 lactylation on PD-L1. **E** The sequences around PD-L1 K280 from the different species were aligned. **F** The lactylation modification sites of PD-L1 were predicted using the FSL-Kla system. **G** IB analysis of the anti-Flag IPs derived from the 293 T cells transfected with the PD-L1-Flag WT, K125R, and K280R mutants.

To identify the lactylation site(s) of PD-L1, we first constructed a PD-L1 intercellular domain (ICD) delated mutant. We found that PD-L1 lactylation was nearly abolished, indicating that the lactylation site(s) resides of PD-L1 within the cytoplasmic domain (Fig. 3A). Furthermore, we substituted each of five lysine (K) residues within the ICD into arginine (R). We found that only the K280R mutant nearly abrogated PD-L1 lactylation (Fig. 3B). We next assessed the influence of lactate on PD-L1 lactylation. We found that lactate administration significantly increased PD-L1 lactylation levels, whereas the PD-L1-K280R-Flag mutant greatly diminished lactylation signals and was unaffected by lactate treatment (Fig. 3C). Additionally, mass spectrometry (MS) analysis also suggested that K280 was the most promising candidate lysine lactylation site (Fig. 3D). A sequence alignment showed that PD-L1 K280 has been highly conserved among different species during evolution (Fig. 3E). FSL-Kla is a few-shot learning-based multi-feature hybrid system for lactylation site prediction [13]. Using this tool, a PD-L1 amino acid sequence analysis was performed. K25 and K280 displayed the highest scores, and they were markedly higher than those of other possible modification sites (Fig. 3F). We therefore constructed a PD-L1-K25R-Flag mutant and found that an unchanged lactylation level compared with PD-L1-WT-Flag (Fig. 3G). Taken together, our results indicate that K280 could be a major PD-L1 lactylation site.

### Lactylation stabilized PD-L1 via controlling its ubiquitination

**Fig. 4:**
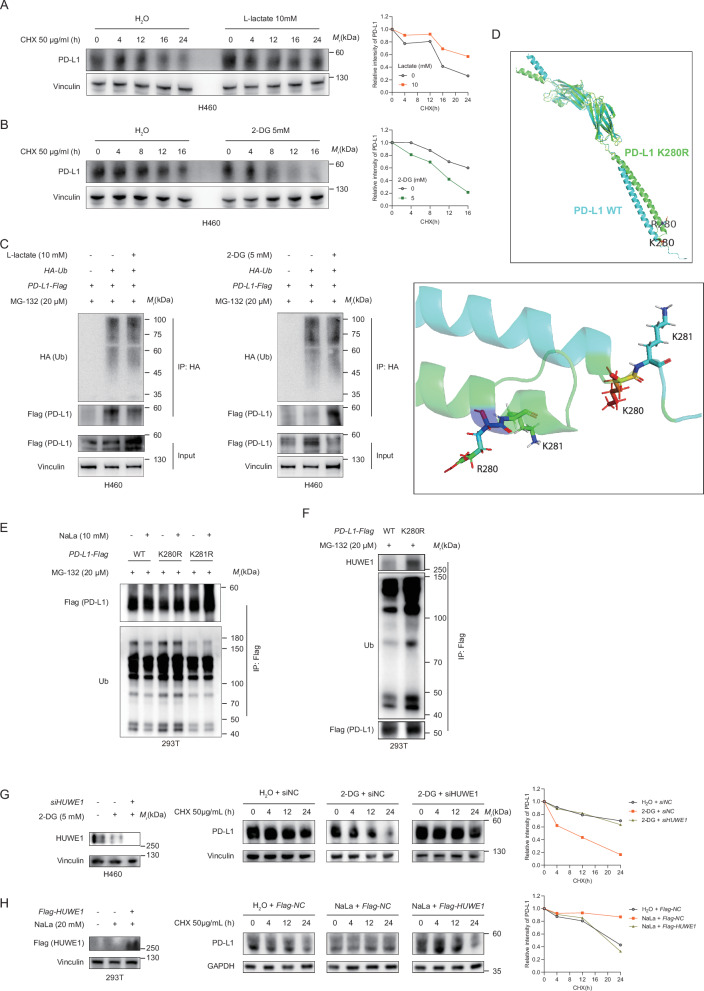
Lactylation stabilized PD-L1 by counteracting its polyubiquitination. **A**, **B** IB analysis of the PD-L1 expression in the H460 cells treated with lactate 10 mM (**A**) or 2-DG 5 mM (**B**) for 24 h and cycloheximide (CHX) at the indicated time points. The PD-L1 protein expression levels were quantitatively estimated based on the western blot analysis. **C** Ubiquitination assay assessing PD-L1 in the 293 T cells co-transfected with HA-Ubiquitin (Ub), and the PD-L1-Flag treated with lactate or 2-DG and MG132. **D** The protein structures of the PD-L1 and PD-L1 K280R mutation predicted by AlphaFold was presented in the cartoon mode (upper panel), and the spatial distribution of K (R) 280 and K281 was rendered in stick mode (bottom panel) using the PyMol software. **E** The Co-IP analysis for the interaction of ubiquitin and PD-L1-Flag in HEK293T cells transfected with PD-L1-Flag (WT, K280R, or K281R) treated with NaLa (10 Mm) for 24 h and MG132 (20 μM) for 6 h. **F** The Co-IP analysis for the interaction of HUWE1, ubiquitin, and PD-L1-Flag in HEK293T cells transfected with PD-L1-Flag (WT or K280R) treated with MG132 (20 μM) for 6 h. **G** Western blot analysis of the PD-L1 expression in the vector control and HUWE1 knockdown H460 cells treated with 2-DG 5 mM for 24 h and cycloheximide (CHX) at the indicated time points. The HUWE1 expression was detected after transfection with siHUWE1 or siNC for 36 h and treated with 2-DG 5 mM mM for 24 h (left panel). The PD-L1 protein expression levels were quantitative estimated based on the western blot analysis (right panel). **H** Western blot analysis of the PD-L1 expression in the empty vector and Flag-HUWE1 overexpression 293 T cells treated with NaLa 20 mM for 24 h and CHX at the indicated time points. The Flag (HUWE1) expression was detected after transfection with Flag-HUWE1 for 36 h and treated with NaLa 20 mM for 24 h (left panel). The PD-L1 protein expression levels were quantitatively estimated based on the western blot analysis (right panel).

Given that lactate could affect PD-L1 expression at the protein level, and PD-L1 was lactylated at lysine 280; hence, we further investigated the mechanism of lactate on PD-L1 expression and whether it was related to lactylation. A cycloheximide (CHX)-chase experiment indicated slower degradation of the PD-L1 protein in the lactate treated H460 cells (Fig. 4A) and faster degradation of the PD-L1 protein in glycolysis inhibitor 2-DG treated cells (Fig. 4B). To elucidate whether lactate modulated PD-L1 degradation occurred through the proteasome pathway, we analyzed PD-L1 ubiquitination in the presence of the proteasome inhibitor MG132. We found that MG132-induced PD-L1 ubiquitination was abolished by L-Lactate administration, and enhanced by 2-DG treatment (Fig. 4C). These results suggested that lactate may enhance PD-L1 protein stability by inhibiting PD-L1 ubiquitination.

To investigate whether PD-L1 K280 lactylation manage the ubiquitination and stability of PD-L1, we further predicted the protein structure of PD-L1 K280R mutation using AlphaFold and found no gross conformational changes due to the PD-L1 K-R mutation at the 280 site (Fig. 4D, upper panel). Furthermore, previous studies have demonstrated that the adjacent amino acid, K281, could be ubiquitinated by the ubiquitin E3 ligase WWE domain-containing protein 1 (HUWE1) [14]. Therefore, we speculated whether PD-L1 lactylated at K280 would hinder E3 ubiquitin ligase binding and K281 polyubiquitination (Fig. 4D, bottom panel). As discussed above, lactate treatment markedly enhanced PD-L1 WT ubiquitination, and overall ubiquitination levels in the K280R mutants were significantly increased. In addition, ubiquitination levels were markedly decreased in the K281R mutants. Furthermore, both mutants did not change under lactate administration (Fig. 4E). The ubiquitination level of PD-L1 and the binding ability between the PD-L1 lactylation dead mutation K280R and HUWE1 was much stronger than that of the PD-L1 WT (Fig. 4F). Moreover, in the H460 cell line, HUWE1 knockdown prolonged the half-life of PD-L1, which was otherwise shortened by the 2-DG treatment (Fig. 4G). Consistently, PD-L1 stabilization induced by NaLa was reversed by HUWE1 overexpression (Fig. 4H). In summary, these findings demonstrated that lactate or lactylation stabilized PD-L1 by counteracting its polyubiquitination by HUWE1 and degradation.

### AARS1 mediates PD-L1 lactylation and influence PD-L1 ubiquitination

**Fig. 5:**
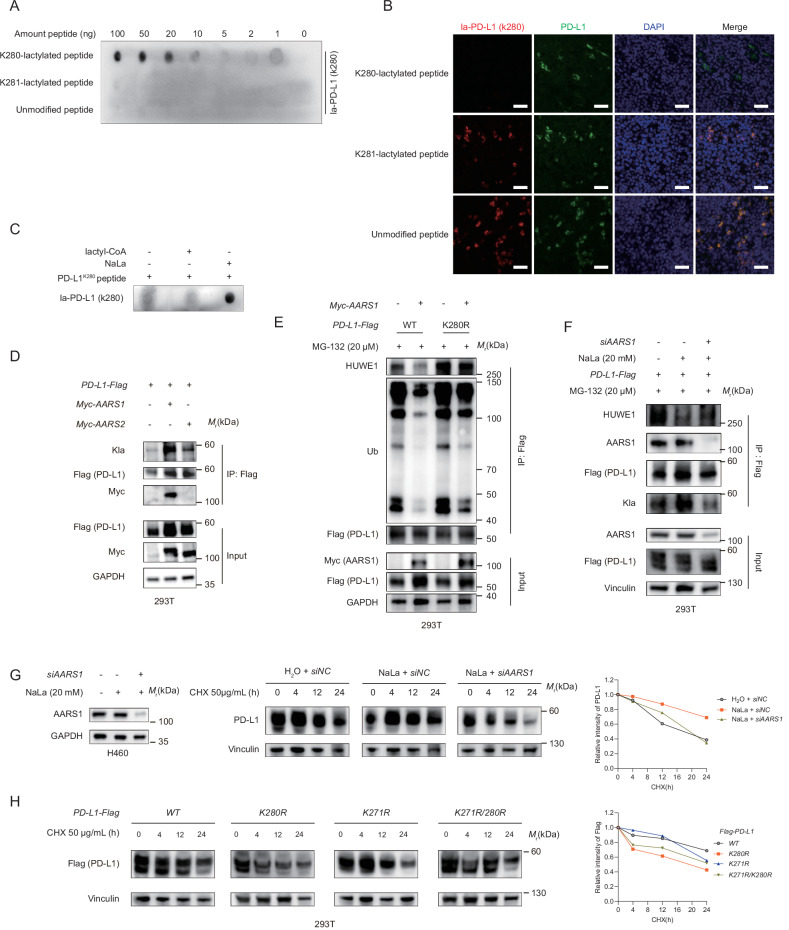
AARS1-mediated lactylation protected PD-L1 from ubiquitination. **A** The specificity of the PD-L1 K280 lactylation antibody was characterized using a dot blot assay. The nitrocellulose membrane was spotted with the K280-lactylated peptide (CQDTNS-(lactyl)K-KQSDTH), the K281-lactylated peptide (CQDTNSK-(lactyl)K-QSDTH), or the unmodified peptide (CQDTNSKKQSDTH) as the indicated amounts and examined using the PD-L1 K280 lactylation antibody. **B** The muti-immunohistochemical (mIHC) specificity of the PD-L1 K280 lactylation antibody was validated using an immunizing peptide blocking experiment. The PD-L1 la-K280 antibody was first neutralized using five times the mass of the peptide overnight and then determined using the mIHC assay. The PD-L1 staining served as a positive control. Scale bar, 20 μm. **C** Immunoblotting with the la-PD-L1 K280 antibody to detect the lactylation of the PD-L1 peptide that contained K280 in vitro in the presence of lactyl-CoA or NaLa. **D** IB analysis of the anti-Flag IPs derived from the 293 T cells transfected with PD-L1-Flag WT and Myc-AARS1 or Myc-AARS2. **E** The Co-IP analysis for the interaction of HUWE1, ubiquitin and PD-L1-Flag in HEK293T cells transfected with Myc-AARS1, PD-L1-Flag (WT or K280R), and treated with MG132 (20 μM) for 6 h. **F** The IB analysis of anti-Flag IPs derived from the 293 T cells transfected with siAARS1 and PD-L1-Flag and treated with NaLa 20 mM for 24 h and MG132 (20 μM) for 6 h. **G** Western blot analysis of the PD-L1 expression in the vector control and AARS1 knockdown H460 cells treated with NaLa 20 mM for 24 h and cycloheximide (CHX) at the indicated time points. The AARS1 expression was detected after transfection with siAARS1 or siNC for 36 h and treated with NaLa 20 mM for 24 h (left panel). The PD-L1 protein expression levels were quantitatively estimated based on the western blot analysis (right panel). **H** Immunoblots of the wild-type (WT) and other mutants of PD-L1 in the 293 T cells following treatment with 50 µg/mL of CHX for the indicated durations. The Flag (PD-L1) protein expression levels were quantitatively estimated based on the western blot analysis.

To directly detect PD-L1 K280 lactylation, we generated an antibody specifically against la-K280 and performed a series of experiments to verify its specificity. A dot blot assay indicated that the PD-L1 laK280 antibody preferentially recognized the K280-lactylated peptide, but not the controlled K281-lactylated peptide or the unmodified peptide. In addition, the signal intensity gradually decreased with a decline in the peptide concentration gradient (Fig. 5A). Multi-immunohistochemistry (mIHC) assays showed that the staining was blocked when the PD-L1 laK280 antibody was preincubated and neutralized with the K280-lactylated peptide as opposed to the controlled K281-lactylated peptide or unmodified peptide (Fig. 5B). However, the specificity of the PD-L1 laK280 antibody used in the western blot assay was poor (data not shown). These data verified the specificity of the PD-L1 laK280 antibody and suggested that the antibody could be used in in vitro assays and mIHC assays.

Previous research has indicated that both lactyl-coenzyme A (lactyl-CoA) and lactate can serve as lactyl-donors, and the corresponding lactyltransferases are acetyltransferase p300 and alanyl-tRNA synthetase1/2 (AARS1/2), respectively [4, 15–17]. To explore the writer and lactyl-donor of PD-L1 lactylation, we first performed an in vitro assay using the PD-L1 peptide in which the amino acid sequence contained K280 as a substrate and NaLa or lactyl-CoA as lactyl-donors. The results showed that NaLa was able to directly lactylate PD-L1 K280 (Fig. 5C). Additionally, the ectopic expression of AARS1, but not AARS2, could bind with PD-L1 and promote PD-L1 lactylation (Fig. 5D). Notably, the ectopic expression of AARS1 clearly disrupted PD-L1 WT ubiquitination and HUWE1 binding; however, the overall ubiquitination level and HUWE1 binding of K280R was markedly elevated and not influenced by AARS1 overexpression (Fig. 5E). Correspondingly, AARS1 knockdown markedly decreased the PD-L1 lactylation level and enhanced its binding to HUWE1, thereby abolishing its stabilization induced by the NaLa treatment (Fig. 5F, G). Recent work by Tong et al. [18] indicated that a serine/glycine-free diet activated HIF-1a, thereby enhancing glycolysis and raising lactate secretion, leading to the lactylation of lysine encoded by the PD-L1 DNA sequence 811–813 (K271). Furthermore, a CHX assay revealed that PD-L1 K271 lactylation delayed degradation through lysosomal pathway. Consistent with this, our mutagenesis analysis indicated that although both the K271R and K280R mutants accelerated PD-L1 degradation individually, the double mutation (K271R/K280R) did not exhibit a synergistic effect (Fig. 5H). These data established AARS1 as a lactyltransferase that directly employs lactate as a lactyl-donor to modify PD-L1 K280 lactylation.

### PD-L1 lactylation promoting tumor immune evasion and cell growth

**Fig. 6:**
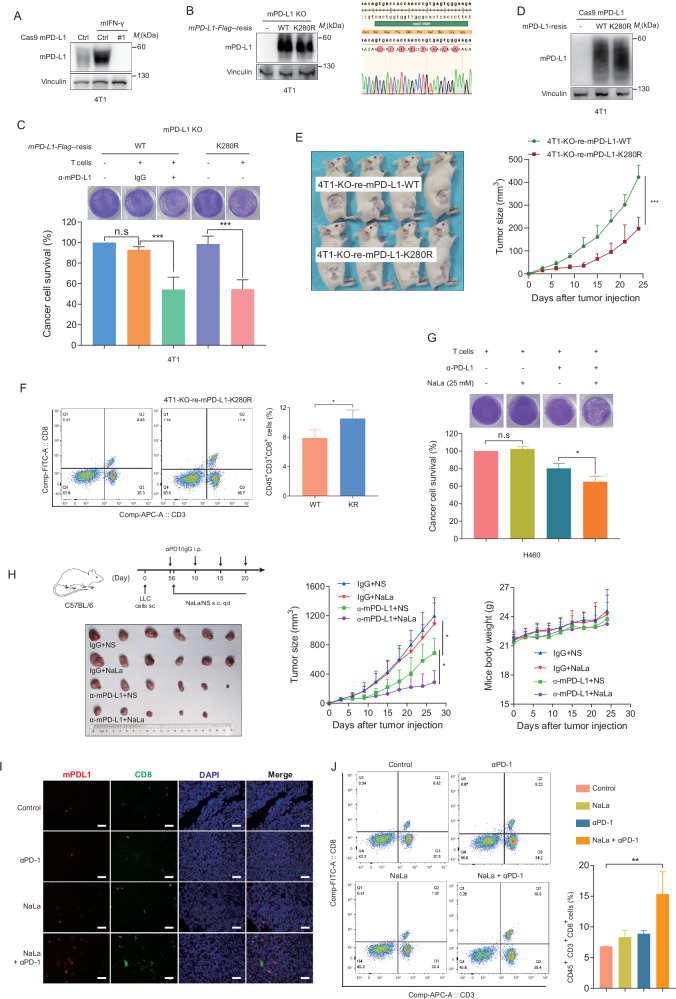
PD-L1 lactylation promoted tumor immune evasion and cell growth. **A** mPD-L1 expression in the 4T1 cells was detected after being transfected with the mPD-L1 Cas9 plasmid and treated with mIFN-γ. **B** mPD-L1 expression in the mPD-L1 knockout (KO) 4T1 cells was detected after being transfected with the mPD-L1-Flag-resis WT or K280R plasmids (upper panel). Sanger sequencing results showed the mPD-L1 Cas9 PAM region of the mPD-L1-Flag-resis WT/K280R plasmids (bottom panel). **C** T cell-meditated tumor cell-killing assay in the mPD-L1 KO 4T1 cells after being transfected with mPD-L1-Flag-resis WT/K280R along with the mPD-L1 antibody or IgG treatment as indicated (upper panel). Quantification of the cell survival rate (bottom panel). The data represent the mean ± SD of three independent experiments, n.s no significant, ***P < 0.001, Student’s t test. **D** The mPD-L1 expression in the 4T1 KO cells with stable mPD-L1-resis-WT/K280R expression was detected by immunoblotting. **E** The 4T1KO-mPD-L1-resis-WT and 4T1KO-mPD-L1-resis-K280R cells were injected into BALB/C mice, and the tumors were measured at the indicated time point. The data represent mean ± SD. **P < 0.01, ***P < 0.001, Student’s t test. **F** The frequency of CD8+ TILs in the 4T1-KO-re-mPD-L1-WT/K280R cells-bearing mice were assessed using flow cytometry. **G** The T cell-meditated tumor cell-killing assay in the H460 cells after treatment with the PD-L1 antibody or NaLa as indicated (upper panel). Quantification of the cell survival rate (bottom panel). The data represent the mean ± SD of three independent experiments, n.s no significant; *P < 0.05, Student’s t test. **H** The tumor growth of Lewis lung cancer cell (LLC) in the C57BL/6 treated with NaLa or the mPD-L1 antibody were monitored at the indicated time point (*n* = 6 mice per group). The data represent the mean ± SD. **P < 0.01, ***P < 0.001, Student’s t test. qd, once daily; sc, subcutaneous administration. **I** Representative images of the mIHC staining for mPD-L1 (red fluorescence) and CD8 (green fluorescence) in mice in the different treatment groups (Scale bar, 20 μm). **J** The frequency of CD8+ TILs in Lewis lung cancer cell-bearing mice treated with the PD-1 antibody and NaLa were assessed using flow cytometry.

To identify the role of lactylation in PD-L1 functions, we first generated 4T1 cells with mPD-L1 knockout (KO) and the re-expression of CRISPR-Cas9 resistance mPD-L1 WT/K280R (Fig. 6A, B). Next, we isolated CD8 + T cells from BALB/C spleens and performed a T-cell killing assay using 4T1KO-mPD-L1-Flag-resis-WT and 4T1KO-mPD-L1-Flag-resis-K280R cells. As shown in Fig. 6C, 4T1KO-mPD-L1-Flag-resis-K280R cells were more sensitive to T cell killing than the 4T1KO-mPD-L1-Flag-resis-WT cells, and the efficacy was comparable to that of the mPD-L1 antibody blockade. We also constructed stable 4T1KO-mPD-L1-resis-WT and 4T1KO-mPD-L1-resis-K280R cells, and the mPD-L1 expression levels were found to be comparable. The cells were then injected into BALB/C mice, and tumor growth was monitored (Fig. 6D). Consistent with the T-cell killing results, the K280R mutation of mPD-L1 greatly impaired 4T1 tumor growth and exhibited more abundant CD8 + T cell infiltration (Fig. 6E, F). This result supported the prediction that PD-L1 lactylation at lysine 280 plays a critical role in attenuating anti-tumor immunity to promote tumor growth.

NaLa promotes PD-L1 stability in vitro; hence, we next performed a T-cell killing assay. We found that NaLa administration remarkably enhanced the efficacy of the PD-L1 blockade (Fig. 6G). Clinically, NaLa Ringer’s solution has been widely used for patient liquid resuscitation and the correction of metabolic acidosis, and its safety is broadly accepted. To investigate the effect of NaLa on the anti-tumor immune response in vivo, C57BL/6 mice were injected with LLC and received a daily subcutaneous (s.c.) administration of NaLa (1.68 g/kg, pH 7.4) beginning on day six. This was performed in combination with intraperitoneal (i.p.) treatment with anti-mPD-L1 (10 mg/kg, day 5 and 20, every five days, as shown in Fig. 6H). As expected, the NaLa treatment had no effect on the tumor growth, but it significantly improved the anti-mPD-1 therapeutic efficacy without notable mouse weight loss (Fig. 1H). The mIHC assay indicated that the mPD-L1 expression level increased in the NaLa and combination group (Fig. 6I). Moreover, an increased amount of CD8^+^ tumor-infiltrating lymphocyte was present in the tumors of the combination group compares to the anti-mPD-L1 monotherapy group (Fig. 6J). Collectively, these results suggested that NaLa or PD-L1 lactylation was able to maintain the PD-L1 expression and confer the therapeutic benefit of immune checkpoint inhibitor (ICIs) therapy.

### Clinical significance of PD-L1 lactylation in NSCLC patient samples

**Fig. 7:**
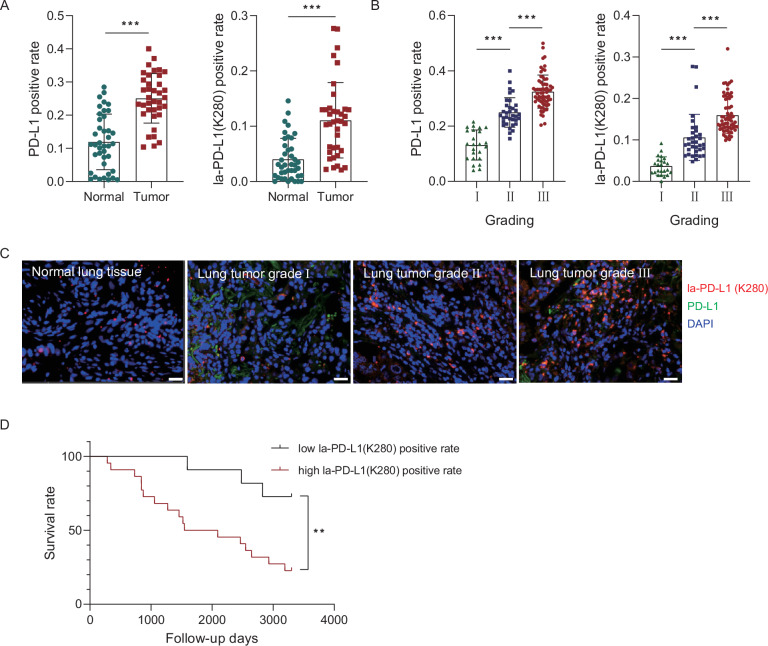
Elevated PD-L1 lactylation was related with an unfavorable prognosis of NSCLC patients. **A** Comparison of the PD-L1 and PD-L1 K280 lactylated rates between normal lung tissue and tumor tissue. **B** The PD-L1 and PD-L1 K280 lactylated rate in NSCLC patients with stages I to III. The P values were calculated using a two-sided Student’s t test. ***P < 0.001. **C** Representative image of the mIHC staining for PD-L1 (green fluorescence) and PD-L1 K280 lactylation (red fluorescence) in normal lung tissues and cancer tissues across different tumor grades of NSCLC patients (Scale bar, 20 μm). DAPI was used as a nuclear marker. **D** The Kaplan–Meier curves of the overall survival durations of the NSCLC patients with low and high PD-L1 K280 lactylated rates in their specimens. The P values were calculated using a log-rank test.

To delineate the clinical significance of PD-L1 K280 lactylation, we conducted a mIHC assay on a tissue microarray comprised of 80 primary NSCLC specimens and paired adjacent normal tissue samples using the PD-L1 laK280 antibody validated by peptide competition (Fig. 5B). We observed that both the PD-L1 and la-PD-L1 (K280) positive rates were markedly higher in the NSCLC samples than in the adjacent normal tissue samples (Fig. 7A). Notably, the levels of the PD-L1 and la-PD-L1 (K280) positive rates were correlated with advanced stages of NSCLC (Fig. 7B). The representative mIHC staining patterns of la-PD-L1 (K280) and the total PD-L1 in normal lung tissue and NSCLC specimens across different tumor grades are shown in Fig. 7C. The survival analysis revealed that the NSCLC patients exhibited high la-PD-L1 (K280) positivity rates. This demonstrated significantly worse overall survival outcomes (Fig. 7D). These results indicated the pivotal role of PD-L1 K280 lactylation in the clinical aggressiveness of NSCLC, making PD-L1 laK280 a potential cancer diagnostic biomarker.

## Discussion

**Fig. 8:**
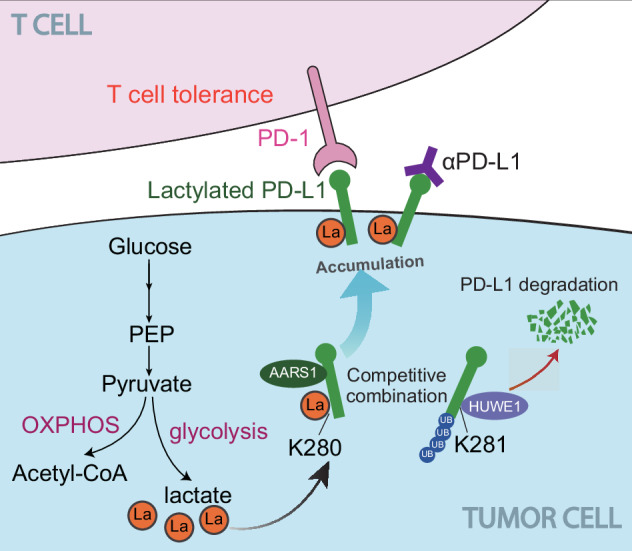
A proposed model illustrating AARS1-coupled PD-L1 K280 lactylation protects HUWE1-mediated PD-L1 degradation and leads to cancer immune escape. Tumor-derived lactate serves as a donor for AARS1-catalyzed PD-L1 lactylation at K280. This modification sterically blocks HUWE1-mediated ubiquitination at the adjacent K281, thereby stabilizing PD-L1. Accumulated PD-L1 engages PD-1 on T cells to drive immune evasion.

The tumor microenvironment is known to regulate PD-L1 expression, but the metabolic pathways that control its stability remain poorly characterized. In this study, we identified lactate as a critical stabilizer of PD-L1 via K280 lactylation modification catalyzed by the lactyltransferase AARS1, which blocked HUWE1-mediated ubiquitination and subsequent proteasomal degradation (Fig. 8). Notably, PD-L1 K280 lactylation correlated with advanced NSCLC progression and worse patient outcomes, suggesting its dual utility as both a therapeutic target and clinical biomarker.

Although lactate has been previously shown to transcriptionally upregulate PD-L1 through the GPR81-TAZ axis [12], we discovered a parallel post-translational mechanism whereby lactate directly stabilized PD-L1 *via* K280 lactylation. While multiple PTMs, including Ser/Thr phosphorylation, palmitoylation, acetylation, glycosylation, and ubiquitination, regulate PD-L1 expression, localization, and ligand binding [7–11], our study focused on lactylation as a novel mechanism governing PD-L1 stability. Beyond PD-L1 regulation, lactate also exhibits pleiotropic immunomodulatory functions [19, 20]. Previous studies have reported that lactate promotes stem-like TCF-1 + CD8 + T cell expansion, a population critical for sustained anti-tumor immunity [21]. Clinically, lactate-based solutions (e.g., Ringer’s lactate) are safely and widely used for fluid resuscitation. In this study, we demonstrated that NaLa administration synergistically enhanced the efficacy of anti-PD-L1 therapy in preclinical models, likely by sustaining PD-L1 expression while preserving CD8 + T cell function. Critically, the lactate-induced stabilization of PD-L1 did not merely enhance immunosuppression, but created a more susceptible target for anti-PD-L1 antibodies, converting an inhibitory signal into an Achilles’ heel in the context of combination therapy. These findings suggest that lactate-enriched formulations could optimize ICI efficacy or improve ex vivo T cell expansion for adoptive therapies such as chimeric antigen receptor T-cell therapy.

A recent study by Tong et al. [18] identified K271 as another lactylation site on PD-L1 that stabilizes the protein by blocking lysosomal degradation. In contrast, we showed that the mutation of K280 to arginine (K280R) significantly reduced but did not abolish PD-L1 lactylation. This result suggested K280 as a major, though not exclusive, modification site. A structural analysis via AlphaFold predicted that the K280R mutation did not disrupt PD-L1’s overall conformation. Mechanistically, K280 lactylation competitively inhibited HUWE1 binding to the adjacent K281 residue, thereby suppressing K281 polyubiquitination and subsequent degradation. Although lactylation at both K280 and K271 of PD-L1 can slow its degradation and operate through distinct mechanisms in tumor cells, they do not exhibit an additive effect. These findings outlined a distinct lactylation-ubiquitination axis in PD-L1 regulation.

The identification of lactyltransferases has evolved significantly since the initial discovery of lactylation [4]. While p300 was initially proposed to utilize lactyl-CoA as a donor, its physiological relevance remains uncertain given the scarcity of intracellular lactyl-CoA (~1000-fold lower than acetyl-CoA) [22]. Recent studies have established AARS1/2-alanyl-tRNA synthetases that exploit the structural mimicry between lactate and alanine as primary lactyltransferases using lactate and ATP as donors [15–17]. Our in vitro assays demonstrated that lactate, not lactyl-CoA, is the donor for PD-L1 K280 modification, and AARS1 is the dominant writer enzyme. Intriguingly, Tong et al. [18] implicated acyltransferase GCN5 as lactyltransferases in PD-L1 K271 lactylation; however, direct evidence for this role remains to be established. Our data suggest that alanine supplementation might antagonize PD-L1 lactylation and offer a strategy to enhance ICI efficacy.

Although lactylation detection of individual proteins typically requires immunoprecipitation, limiting the clinical applicability, we developed a specific antibody for PD-L1 K280 lactylation detection in patient tissues. Our mIHC analysis revealed that PD-L1 lactylation predicted the ICI response and prognosis in NSCLC, offering a practical biomarker for patient stratification.

In summary, our study elucidated a lactate-PD-L1 regulatory axis wherein AARS1-mediated lactylation stabilized PD-L1 to foster immune evasion. By linking tumor metabolism to immune checkpoint control, these findings advocate for targeting lactylation to augment immunotherapy. Further exploration of lactate-based adjuvants and lactylation inhibitors may unlock novel therapeutic avenues for cancer treatment.

## Methods

### Cell culture and transfection

The NSCLC cell lines H460, H1975, H1299, embryonic kidney HEK293T, murine LLC, and the breast cancer line 4T1 were purchased from the American Tissue Culture Collection. The human normal bronchial epithelial 16HBE cells were purchased from Lonza Clonetics (Walkersville). The cells above were cultured in Dulbecco’s Modified Eagle Medium or PRMI 1640 medium supplemented with 10% fetal bovine serum (HyCyte) at 37°C in a 5% CO2 incubator. Transient transfection of the plasmids and siRNAs was conducted using the Lipofectamine 2000 Kit (Invitrogen).

### Plasmids construction and RNA interference

The shGPR81 RNA was constructed in the pLKO.1 vector, and the mature antisense sequence was as follows: 5’-CTGCTAGACTCTATTTCCT-3’. The siRNA sequences used were as follows: for AARS1, 5′-GGUGGAUGACAGCAGUGAAGA-3′; for HUWE1, 5′-CAC ACC AGC AAT GGC TGC CAG AAT T-3′. The PCR amplified human PD-L1 full-length or C-tail deletion mutant (amino acids 263–290, ΔICD), mouse PD-L1 (mPD-L1), and AARS1/2 were cloned into the pcDNA3.1-Flag or pcs2-HA vector. In the HA-ins-PD-L1, the HA-tag was inserted into the PD-L1 sequence after the signal peptide sequence. The PD-L1-Flag mutants, including K263R, K270R, K271R, K280R, K281R, and K280R/K281R, were generated using a Quik Change site-directed mutagenesis kit (Stratagene). sgRNA targeting of mPD-L1 was subcloned into the LentiCRISPRv2 vector, and the sequence was as follows: 5′-GTGACCACCAACCCGTGAGT-3’. The sgRNA-resistant mPD-L1 constructs (Cas9-res-mPD-L1) were synthesized by introducing nonsense mutations in the PAM targeting sites (Fig. 6B). The core plasmids used to generate the Cas9-resistant mPD-L1 (WT and K280R) stable expression cell lines were cloned into pLVX-IRES-Neo vectors.

### CRISPR/Cas9-mediated gene knockout

The stable mPD-L1 KO in the mouse cell line 4T1 was generated using lentiCRISPR v2 vectors that contained mPD-L1 sgRNA produced in 293 T cells and selected with puromycin for 5 to 7 days. The mPD-L1 expression was identified by western blotting.

### Generation of the stable cell lines

Stable GPR81 KD in the H1975 cells was produced using lentiviruses produced in 293 T cells with packaging plasmids (psPAX2), an envelope plasmid (pMD2.G), and pLKO.1 vectors that contained GPR81 shRNA. The cells were infected and selected with puromycin for 5 to 7 days, and the GPR81 expression was detected by western blotting.

The stable Cas9-res-mPD-L1 overexpression 4T1 cells were generated using lentiviruses produced in 293T cells with packaging plasmids (psPAX2), an envelope plasmid (pMD2.G), and pLVX-IRES-Neo vectors that contained Cas9-res-mPD-L1. The 4T1 cells were then infected and selected with puromycin for 5 to 7 days, and the mPD-L1 expression was evaluated by western blotting.

### Western blotting and co-immunoprecipitation (co-IP)

The procedures for western blotting and co-IP were performed as previously described [23]. Detailed information regarding the antibodies used in this study is listed in Table S1.

### Quantitative real-time reverse transcriptase PCR (qRT-PCR)

The total RNA was isolated using the TRIzol reagent (Invitrogen) according to the manufacturer’s instructions [24]. The cDNA was synthesized using the HiScript® III 1st Strand cDNA Synthesis Kit with the gDNA wiper (Vazyme), and qRT–PCR was conducted using the ChamQ Universal SYBR qPCR Master Mix (Vazyme). The primer sequences for the quantitative realtime reverse transcripatase PCR were as follows: PD-L1 forward, 5’- ACTGGCATTTGCTGAACGC-3’; PD-L1 reverse, 5’- ACAATTAGTGCAGCCAGGTCT-3′; GPR81 forward, 5’-AATTTGGCCGTGGCTGATTTC-3’; GPR81 reverse, 5’- ACCGTAAGGAACACGATGCTC-3’; actin forward, 5′-GCAAAGACCTGTACGCCAACA-3′; and actin reverse, 5′-TGCATCCTGTCGGCAATG-3′.

### Antibody generation

A polyclonal antibody specific for PD-L1 K280 lactylation was developed by PTM BIO (Hangzhou, China) targeting the lactylated lysine residue at position 280 within the PD-L1 sequence. The immunization antigen consisted of a synthetic peptide (CQDTNS-(lactyl)K-KQSDTH) that contained the lactylated K280 modification. Antibody specificity was rigorously validated using the following: (1) a dot blot analysis that compared the reactivity against the target peptide versus control peptides (K281-lactylated: CQDTNSK-(lactyl)K-QSDTH; unmodified: CQDTNSKKQSDTH), and (2) peptide blocking experiments where the pre-incubation with immunizing peptide abolished the signal detection.

### In vitro lactylation assay

An in vitro lactylation assay was performed according to previous research [15]. In brief, the reaction mixtures containing 20 mM KCl, pH 7.6, 10 mM MgCl2, 50 mM Tris HCl, and 0.5 mg/ml PD-L1 peptide (CQDTNSKKQSDTH) with 10 mM NaLa, 10 mM ATP, or lactyl-CoA was incubated at 37°C for 1 h. The protein samples were then spotted at the nitrocellulose membrane, naturally air dried, and examined using the PD-L1 K280 lactylation antibody.

### T cell-mediated tumor cell killing assay

CD8^+^ T cells were isolated from either the BALB/c mouse spleens or human PBMCs via magnetic bead isolation. Cells were activated in anti-CD3-precoated plates (10 μg/mL, overnight) with RPMI 1640 containing 5 μg/mL anti-CD28 antibody (BioLegend) and 2 ng/mL IL-2 (PeproTech) for 72 hr. IFN-γ-pretreated (50 ng/mL, 24 hr) 4T1 or H460 tumor cells were co-cultured with activated T cells at a 10:1 effector-to-target ratio in the presence of the IgG control (10 μg/mL), anti-PD-L1 antibody (10 μg/mL), or NaLa (40 mM). After 72 h, the viable tumor cells were quantified using crystal violet staining (0.1% w/v) followed by solubilization in 10% glacial acetic acid and OD570 measurement.

### Animal studies

Female C57BL/6 and BALB/c mice (6–8-weeks old) were purchased from the Vital River Laboratory Animal Technology Co., Ltd. (Beijing, China). All animal procedures were performed according to the guidelines approved by Zhejiang University. The BALB/C mice were injected subcutaneously with 1 × 10^6^ 4T1KO-mPD-L1-resis-WT and 4T1KO-mPD-L1-resis-K280R cells in 50 μL of medium mixed with 50 μL of Matrigel (BD) on day 0. The tumor volume was measured every 3 days until day 25. LLC cells 1 × 10^6^ in 100 μL of medium were injected subcutaneously into the C57BL/6 mice and after 5 days. The mice were intraperitoneally injected with 250 μg anti-mPD-1 antibody (BE0146, Bio X Cell) or IgG control (BE0089, Bio X Cell) every 5 days as indicated and subcutaneously injected with NaLa or normal saline 1.68 g/kg daily. The tumor volumes were calculated using the formula: length × width^2^ × 0.5.

### Tumor sample preparation and flow cytometry

The excised tumors were digested using collagenase/DNase at 37°C for 1 h. Single-cell suspensions were generated by filtering the digested samples through 70-μm strainers (BD Biosciences), followed by centrifugation and erythrocyte lysis using an ammonium-chloride-potassium (ACK) buffer (Beyotime Biotechnology). Cells were stained with FITC-CD8 and APC-CD3 antibodies (BioLegend). Flow cytometry was performed on a Beckman Coulter CytoFLEX platform, with data analysis using CytExpert V2.3 software.

### Multiplex immunohistochemistry

Formalin-fixed paraffin-embedded sections from murine tumor tissues and human NSCLC tissue microarrays (LAC-202204, recordbio, Shanghai) underwent multiplex staining. After standard deparaffinization and rehydration, antigen retrieval was performed in an ethylenediaminetetraacetic acid (EDTA) buffer (pH 9.0). Endogenous peroxidase was blocked with 3% H_2_O_2_ (15 min), followed by protein blocking with 3% BSA/PBST (1 h). Primary antibodies against specific targets (detailed antibody information is provided in the Supplementary Table S1) were incubated overnight at 4°C. Detection was achieved through biotinylated secondary antibodies and streptavidin-HRP conjugation, followed by Sulfo-Cyanine3 incubation. Antibody complexes were stripped via citrate-based antigen retrieval (pH 6.0) prior to repeating the staining cycle with species-matched secondary antibodies conjugated to spectrally distinct fluorophores (FITC). Nuclei were counterstained with 4′,6-diamidino-2-phenylindole (DAPI), and the sections were mounted in a glycerol-based medium. Imaging was performed using a laser-scanning confocal microscope.

## Supplementary information

Figure S1

Supplementary figure legends

Table S1

original WB

## Data Availability

Uncropped western blots performed in this study are included in this publication. Other figures or data supporting the results of this study are available from the corresponding author upon reasonable request.
