# Supplementary figures and images for: Lactylation stabilizes PD-L1 to promote tumor immune evasion and cell growth
Source: Cell Death Dis. 2026 Mar 21;17(1):335. doi: 10.1038/s41419-026-08589-1 (PMC13039446; doi:10.1038/s41419-026-08589-1)

A

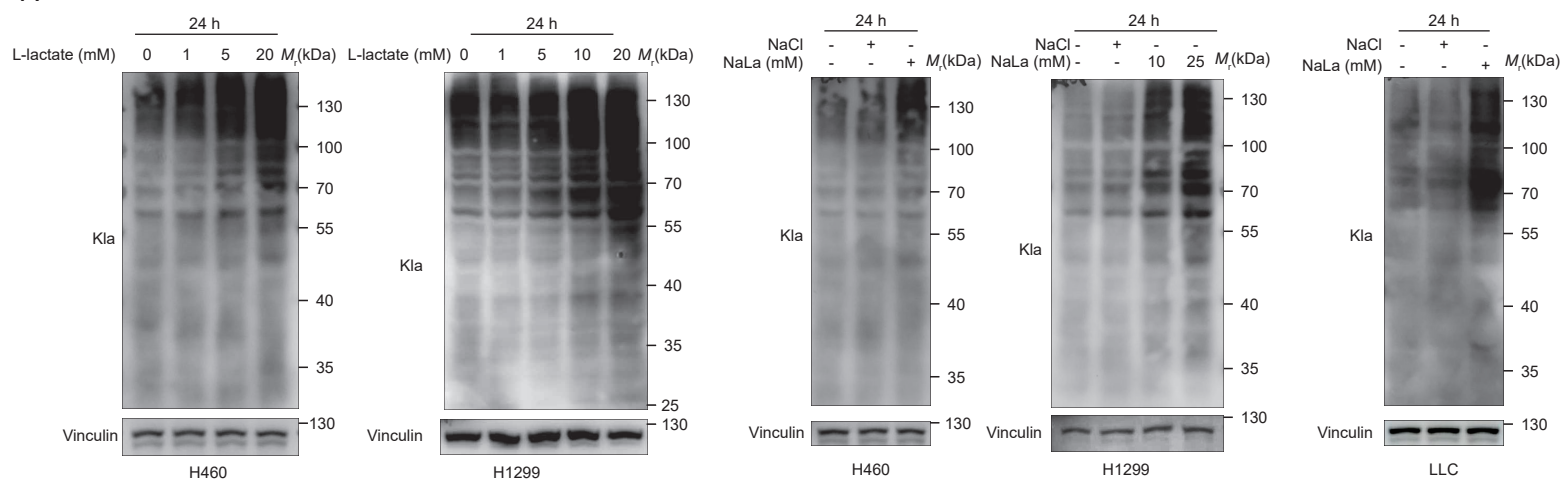

B

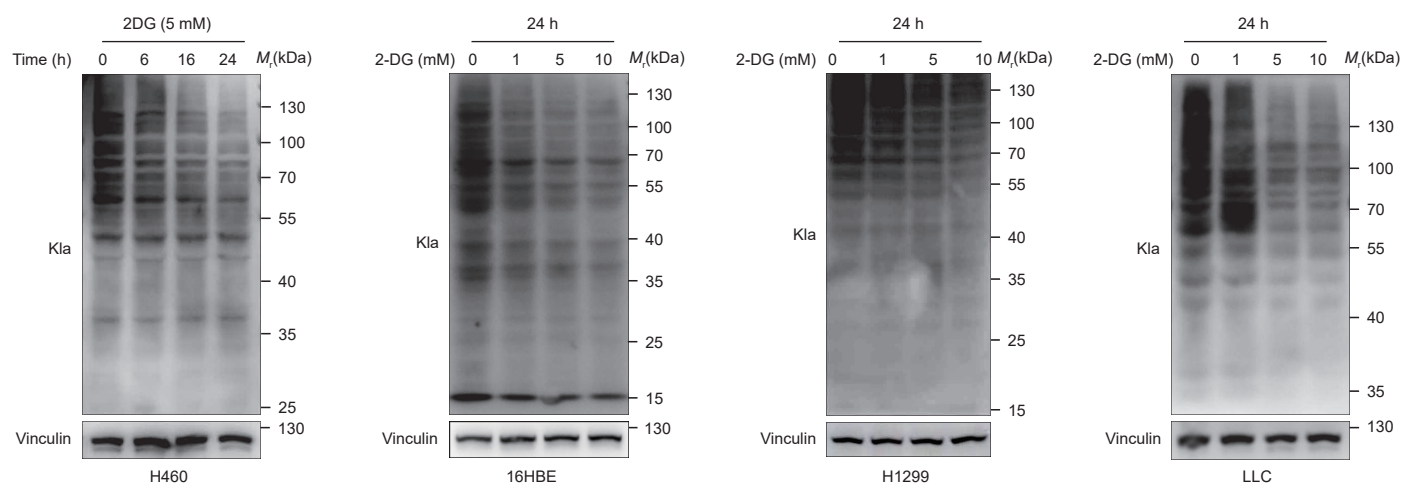

C

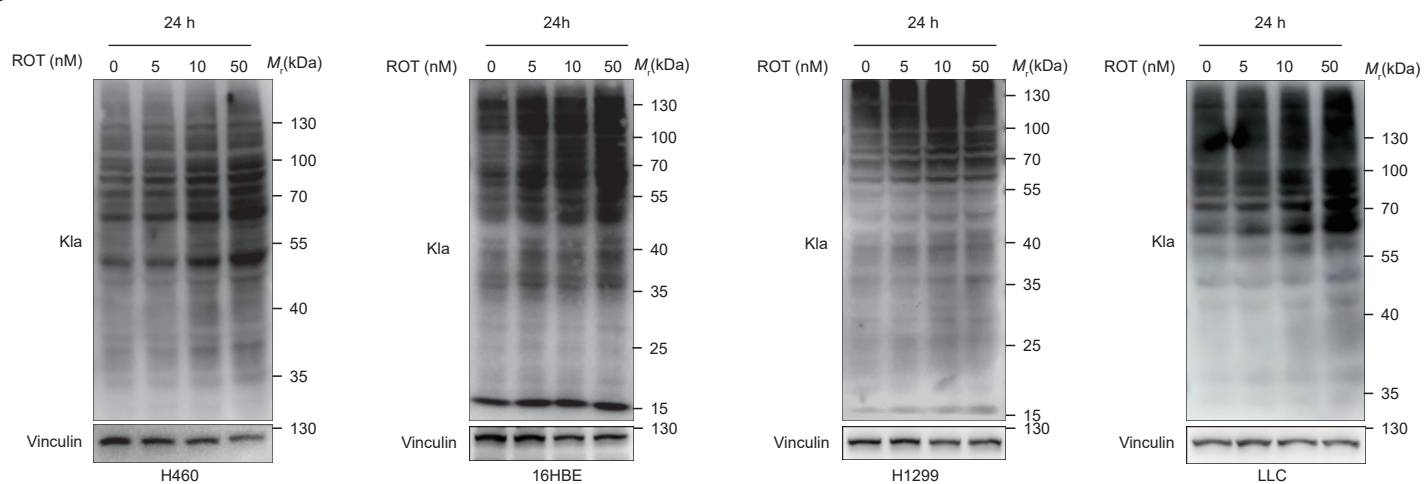

Supplement: Supplementary file 1 — Figure S1 [file 41419_2026_8589_MOESM1_ESM.pdf]
