# Supplementary material for: Lactylation stabilizes PD-L1 to promote tumor immune evasion and cell growth
Source: Cell Death Dis. 2026 Mar 21;17(1):335. doi: 10.1038/s41419-026-08589-1 (PMC13039446; doi:10.1038/s41419-026-08589-1)
Supplement: Supplementary file 2 — Supplementary figure legends [file 41419_2026_8589_MOESM2_ESM.docx]

**Fig. S1 Non-histone proteins could be lactylated.** Western blot results using a pan-lactylated antibody of 16HBE or different histological non-small-cell lung cancer cell lines after treated with lactate or NaLa (A), 2-deoxy-d-glucose (2-DG, B) or rotenone (ROT, C) as indicated. Kla, lactylated lysine.
