# Supplementary material for: Lactylation stabilizes PD-L1 to promote tumor immune evasion and cell growth
Source: Cell Death Dis. 2026 Mar 21;17(1):335. doi: 10.1038/s41419-026-08589-1 (PMC13039446; doi:10.1038/s41419-026-08589-1)
Supplement: Supplementary file 4 — original WB [file 41419_2026_8589_MOESM4_ESM.pptx]

## Slide 1
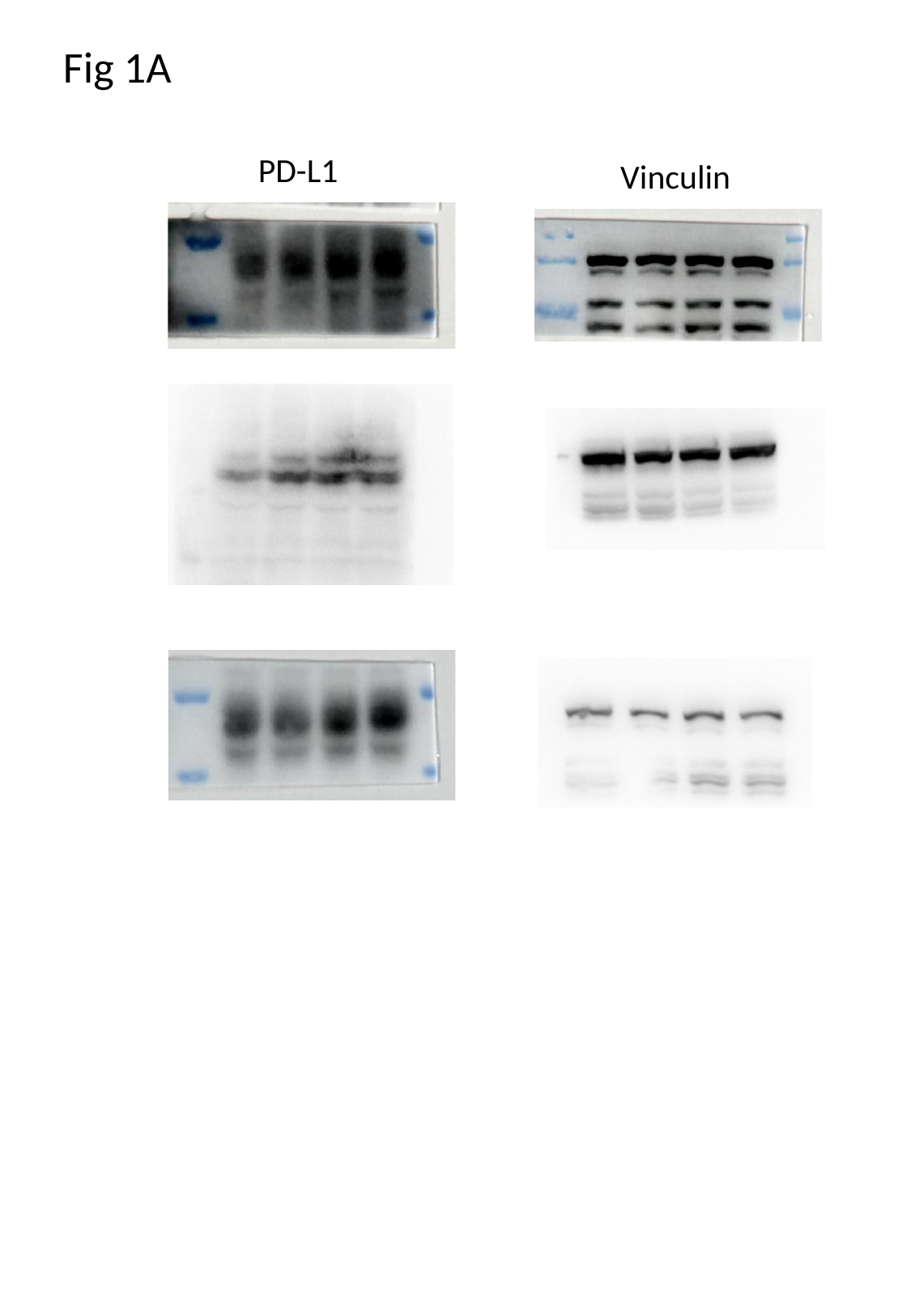

Fig 1A
PD-L1
Vinculin

## Slide 2
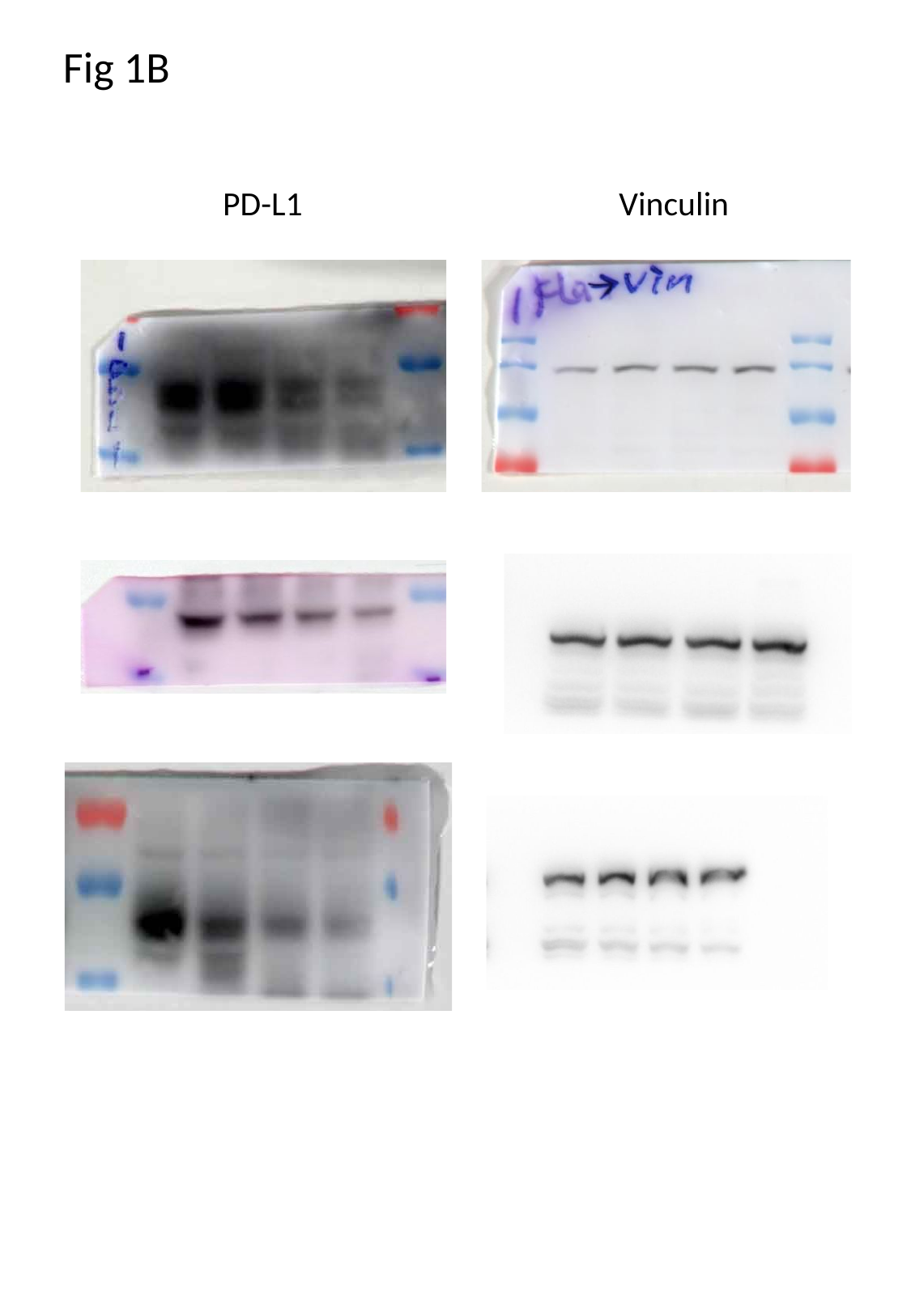

Fig 1B
PD-L1
Vinculin

## Slide 3
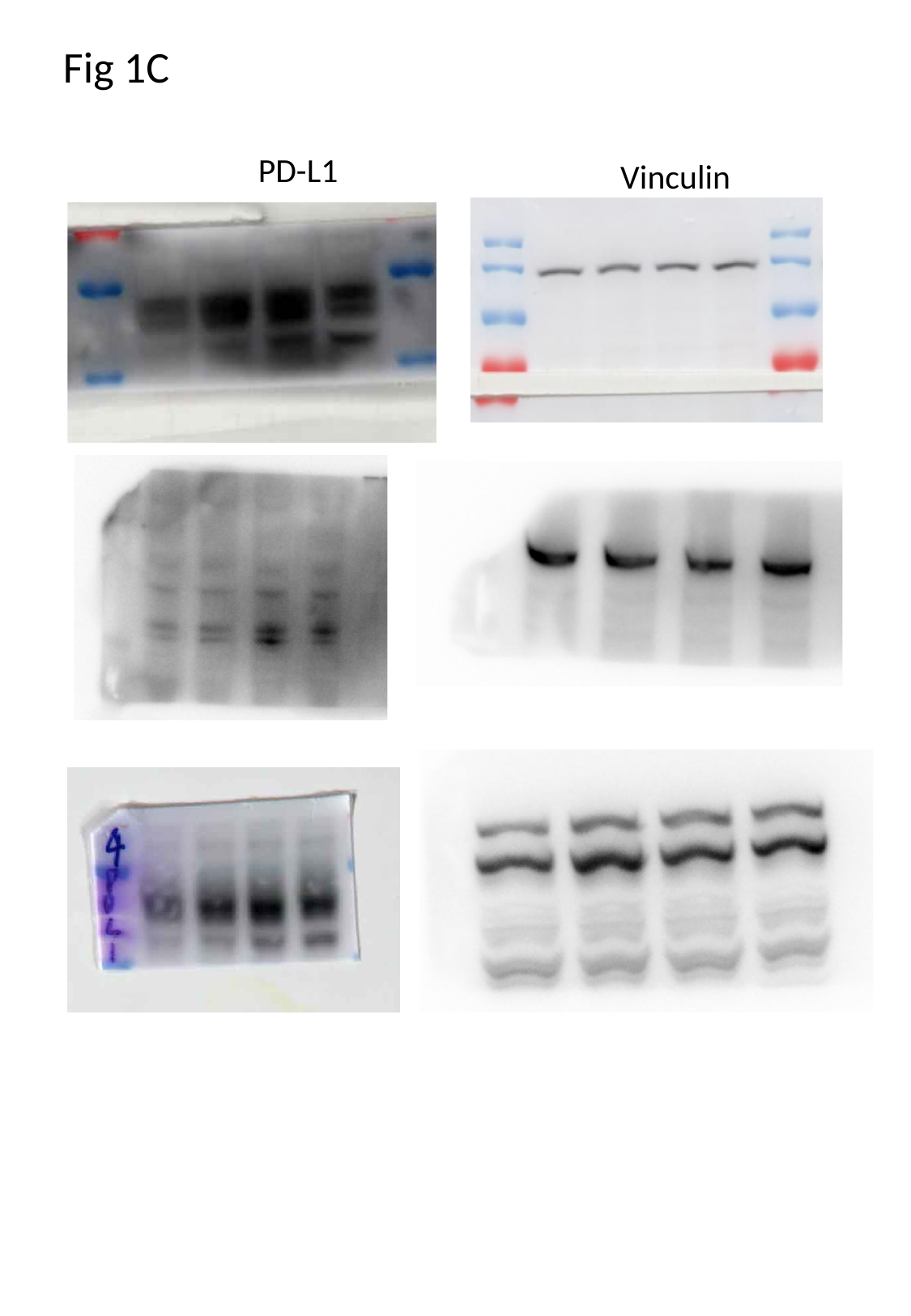

Fig 1C
PD-L1
Vinculin

## Slide 4
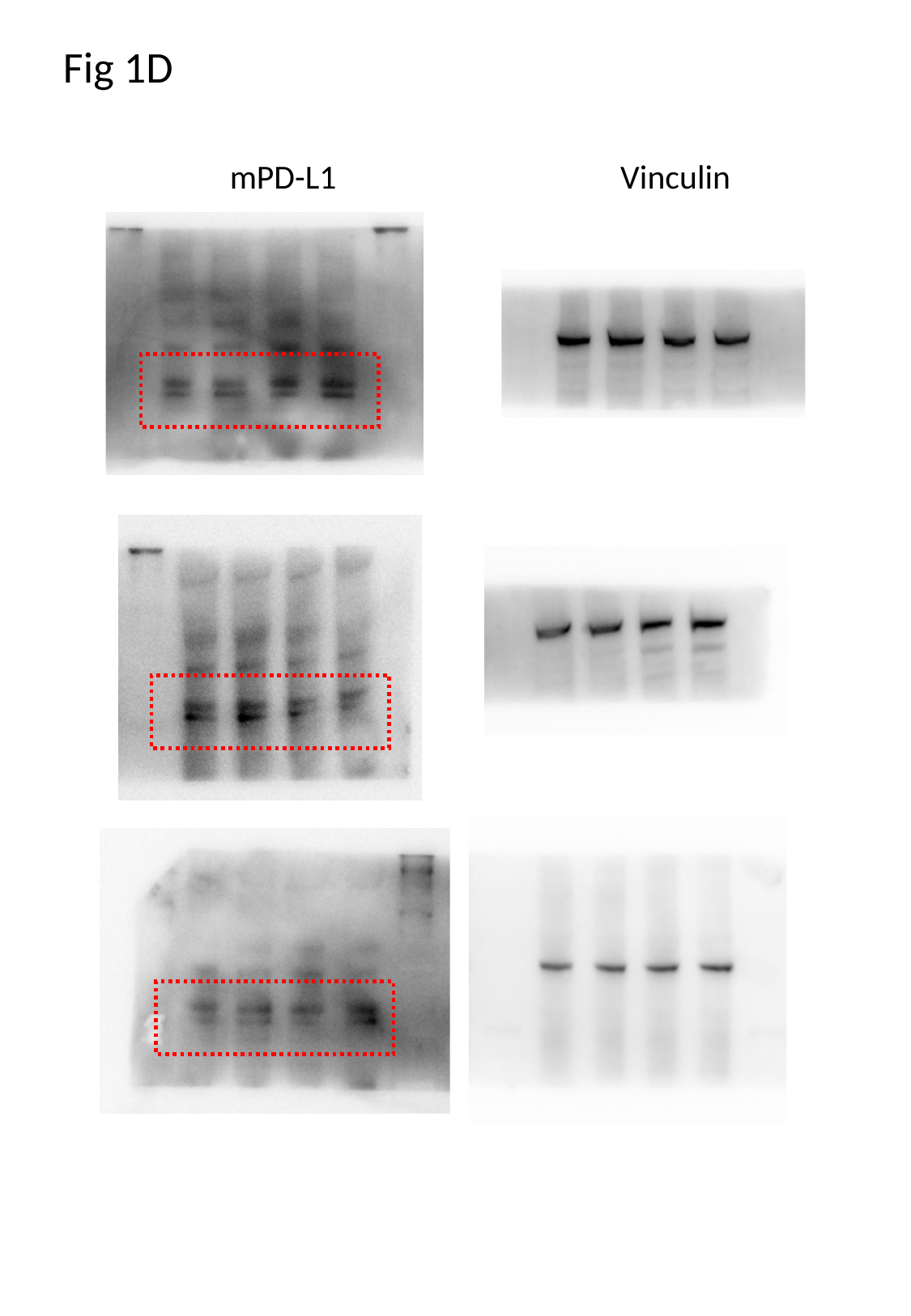

Fig 1D
mPD-L1
Vinculin

## Slide 5
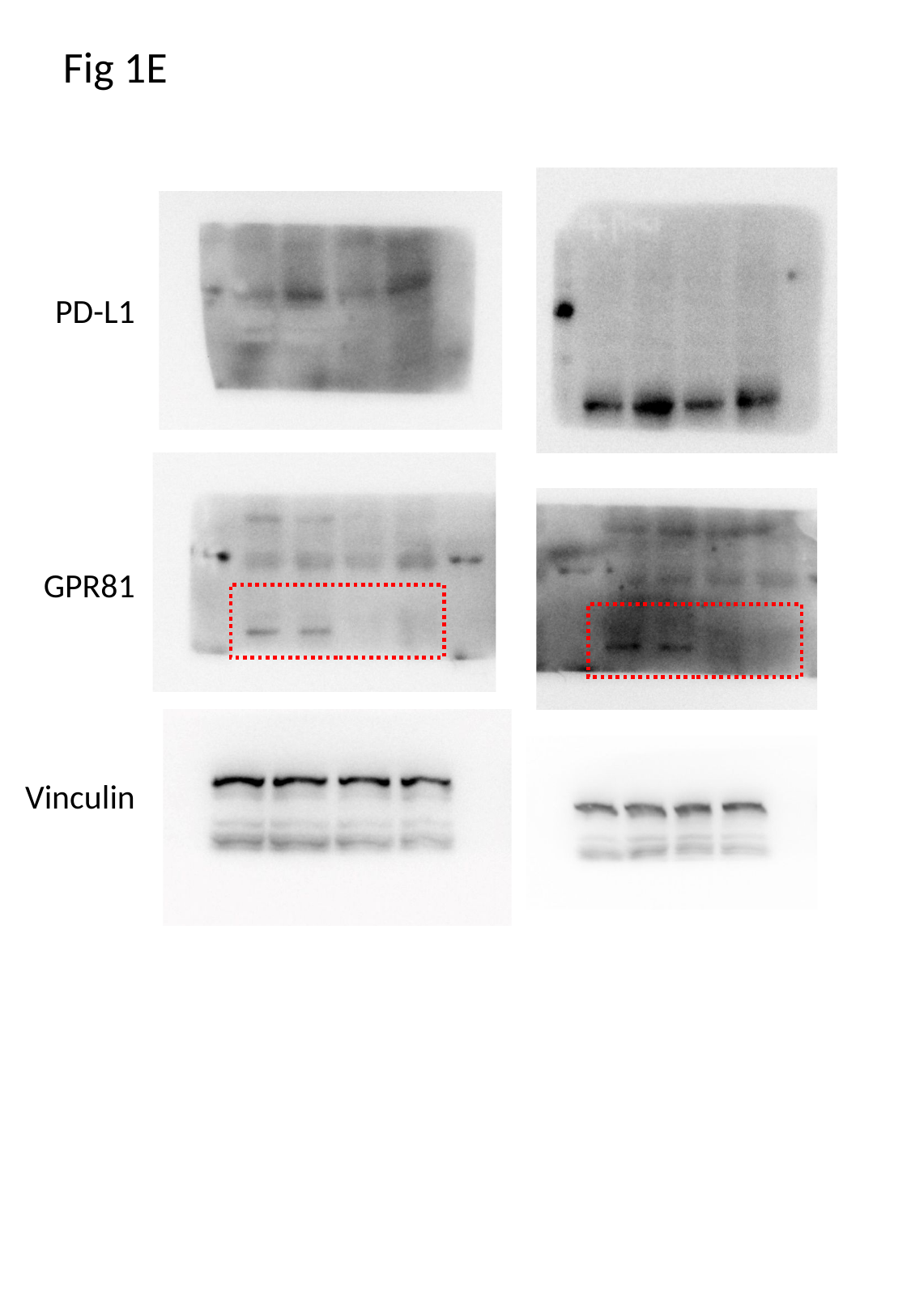

Fig 1E
PD-L1
GPR81
Vinculin

## Slide 6
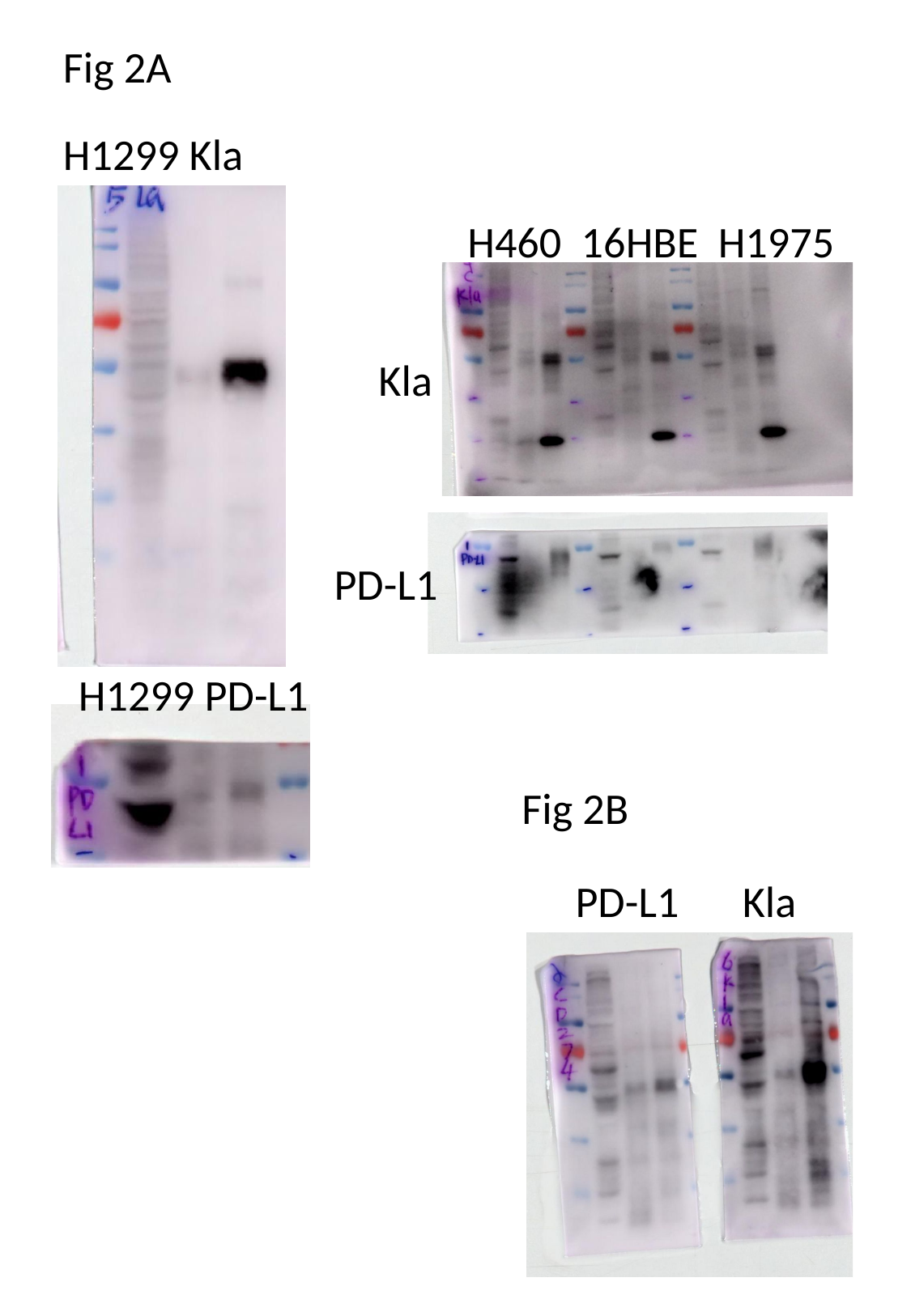

Fig 2A
H1299 Kla
H460 16HBE H1975
Kla
PD-L1
H1299 PD-L1
Fig 2B
PD-L1
Kla

## Slide 7
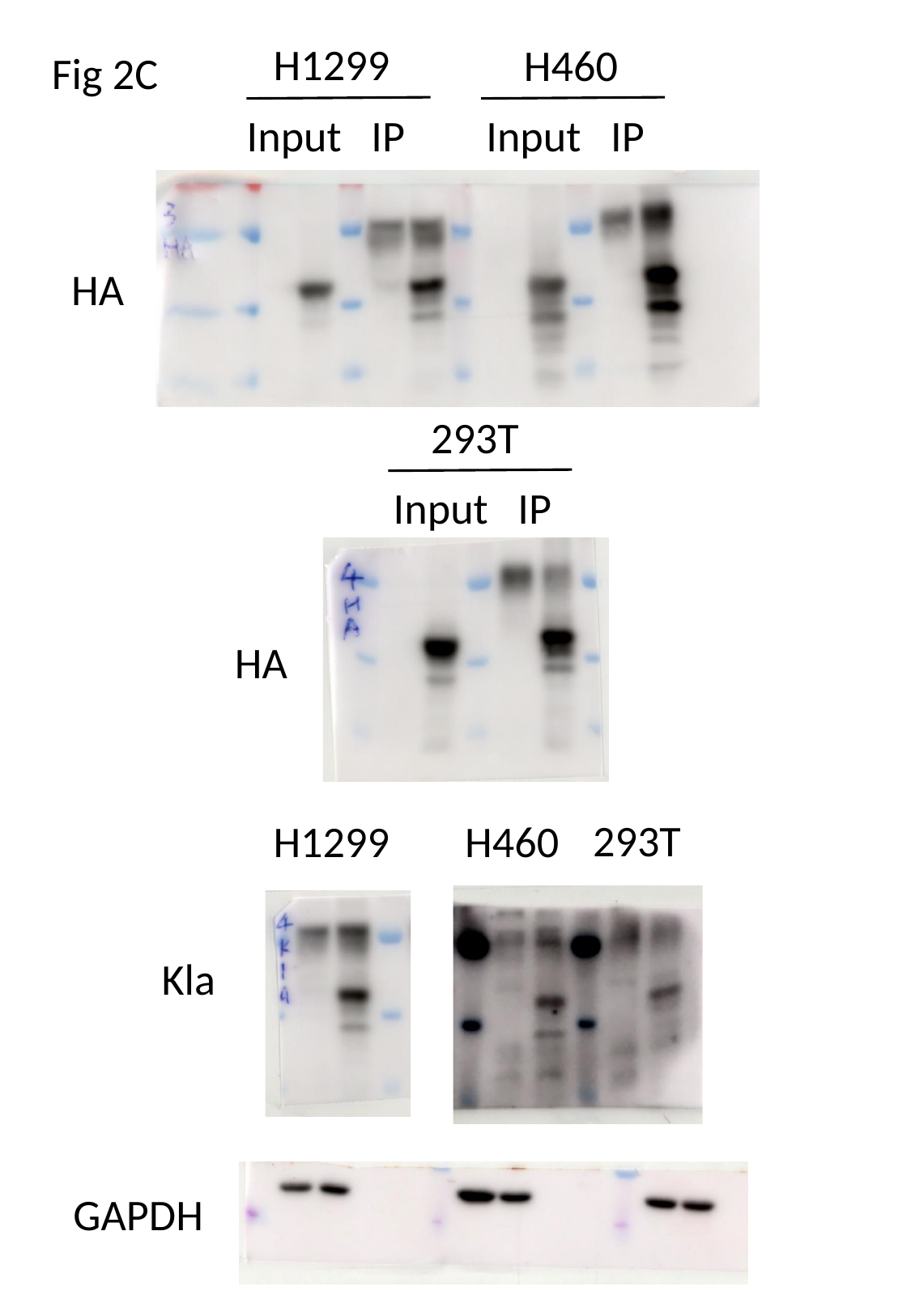

H1299
H460
Fig 2C
Input IP
Input IP
HA
293T
Input IP
HA
293T
H1299
H460
Kla
GAPDH

## Slide 8
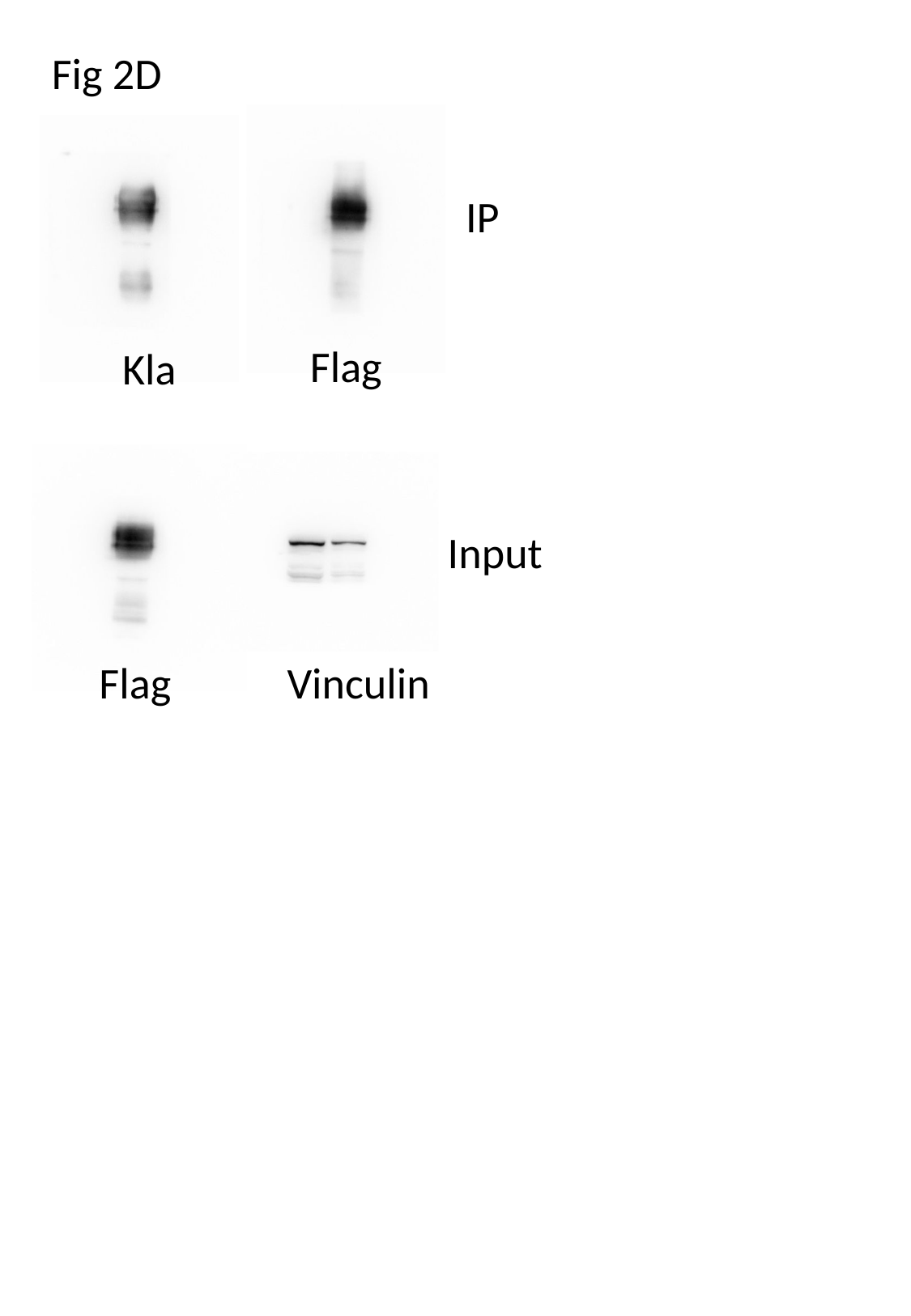

Fig 2D
IP
Flag
Kla
Input
Vinculin
Flag

## Slide 9
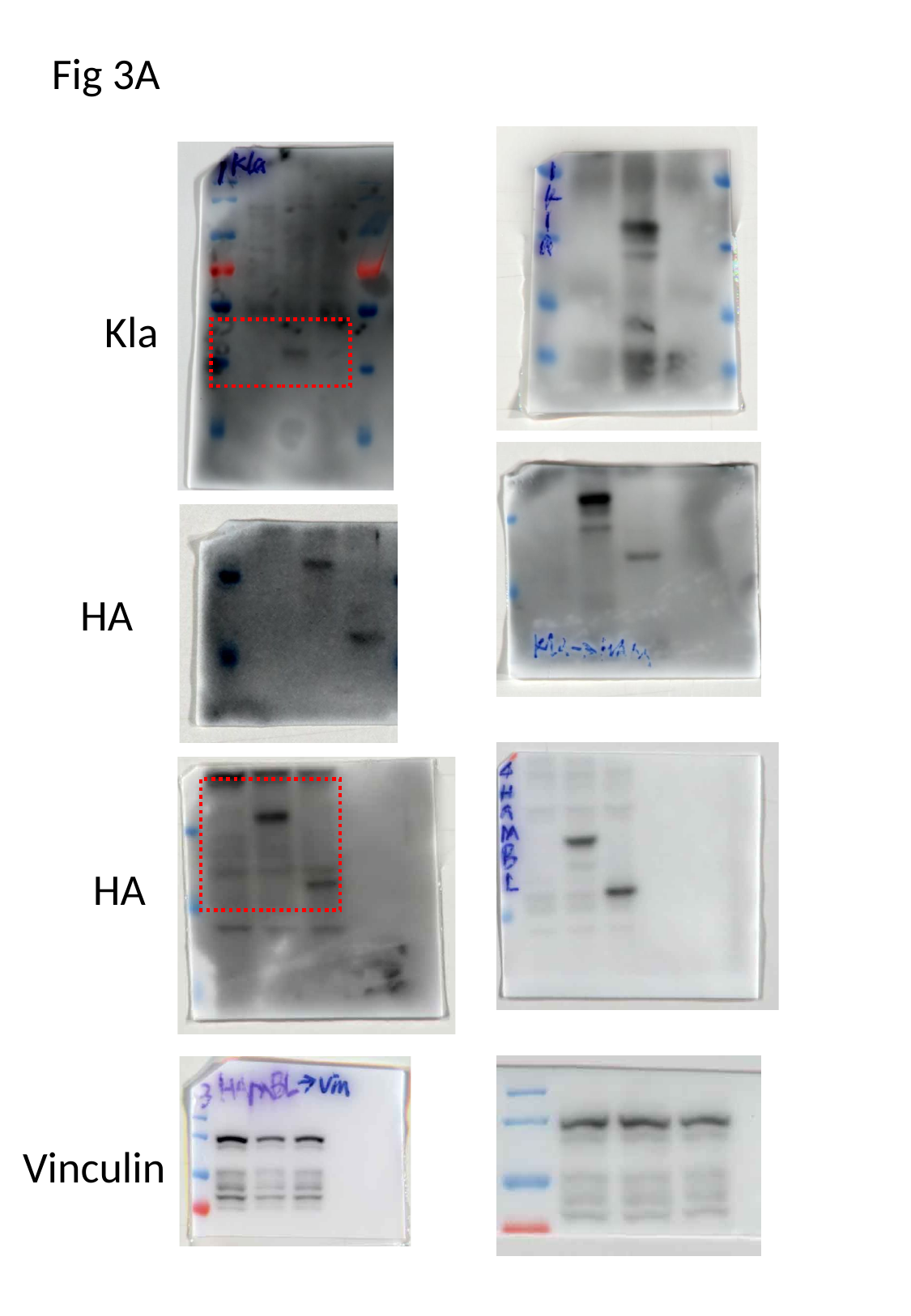

Fig 3A
Kla
HA
HA
Vinculin

## Slide 10
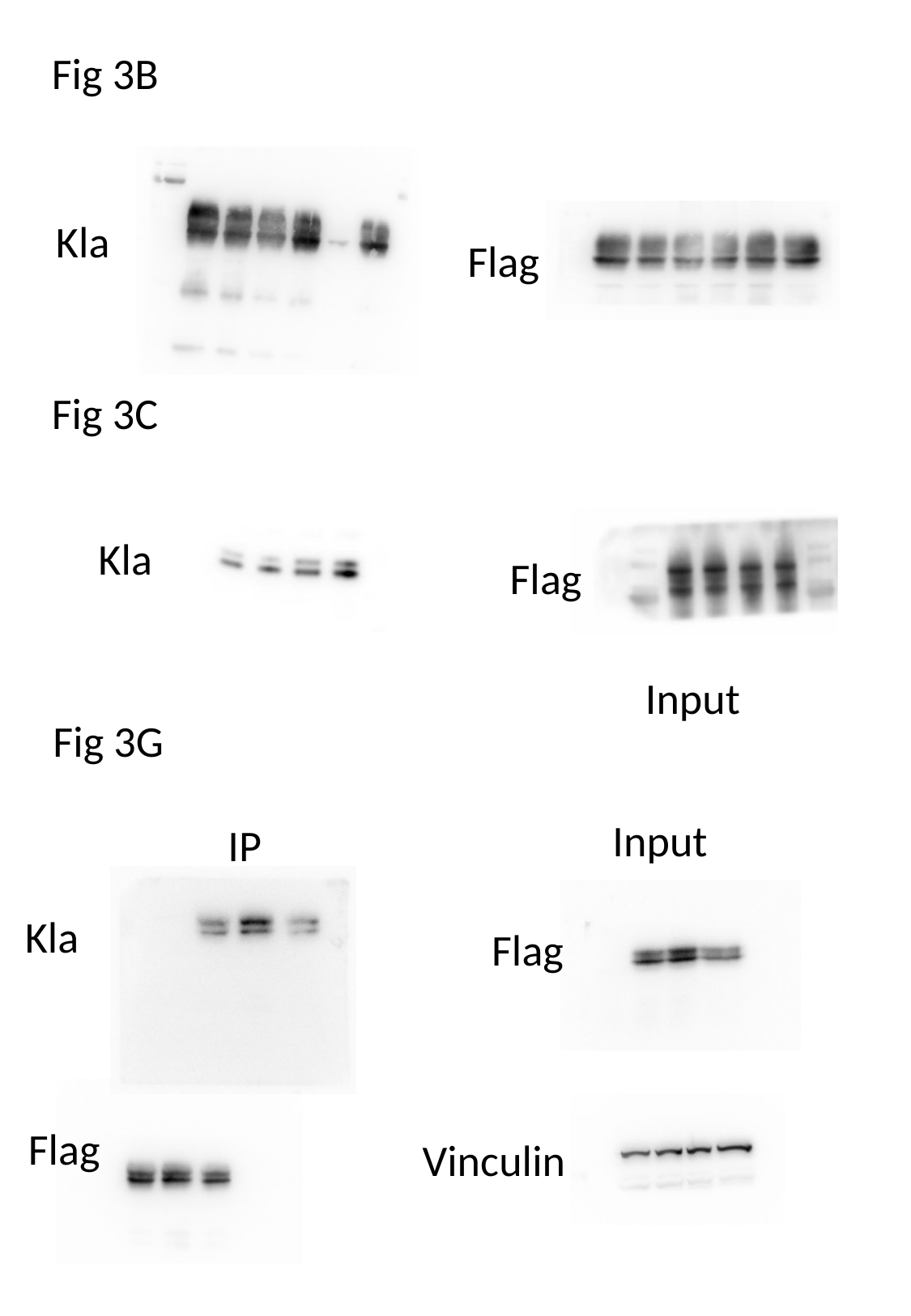

Fig 3B
Kla
Flag
Fig 3C
Kla
Flag
Input
Fig 3G
Input
IP
Kla
Flag
Flag
Vinculin

## Slide 11
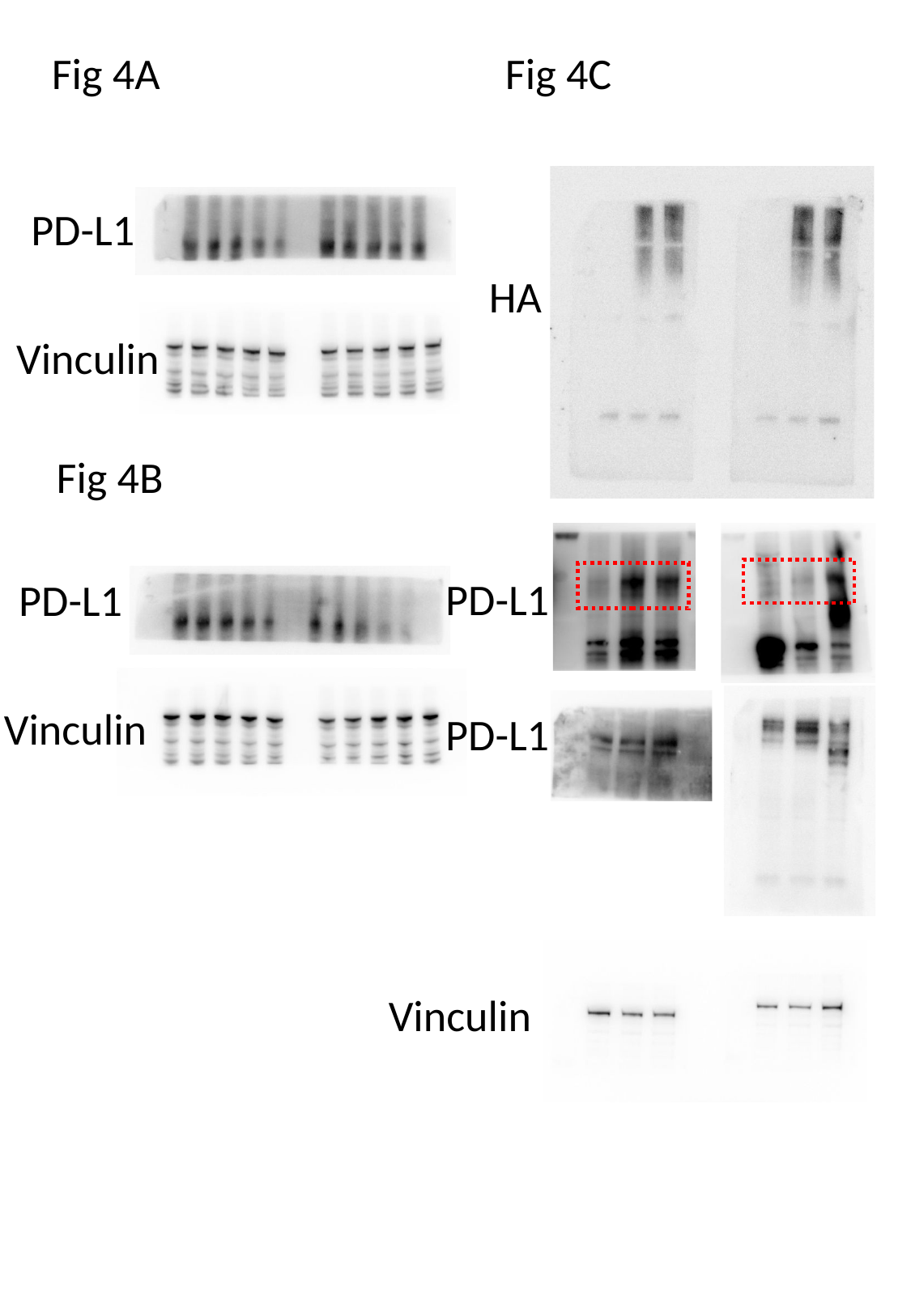

Fig 4A
Fig 4C
PD-L1
HA
Vinculin
Fig 4B
PD-L1
PD-L1
Vinculin
PD-L1
Vinculin

## Slide 12
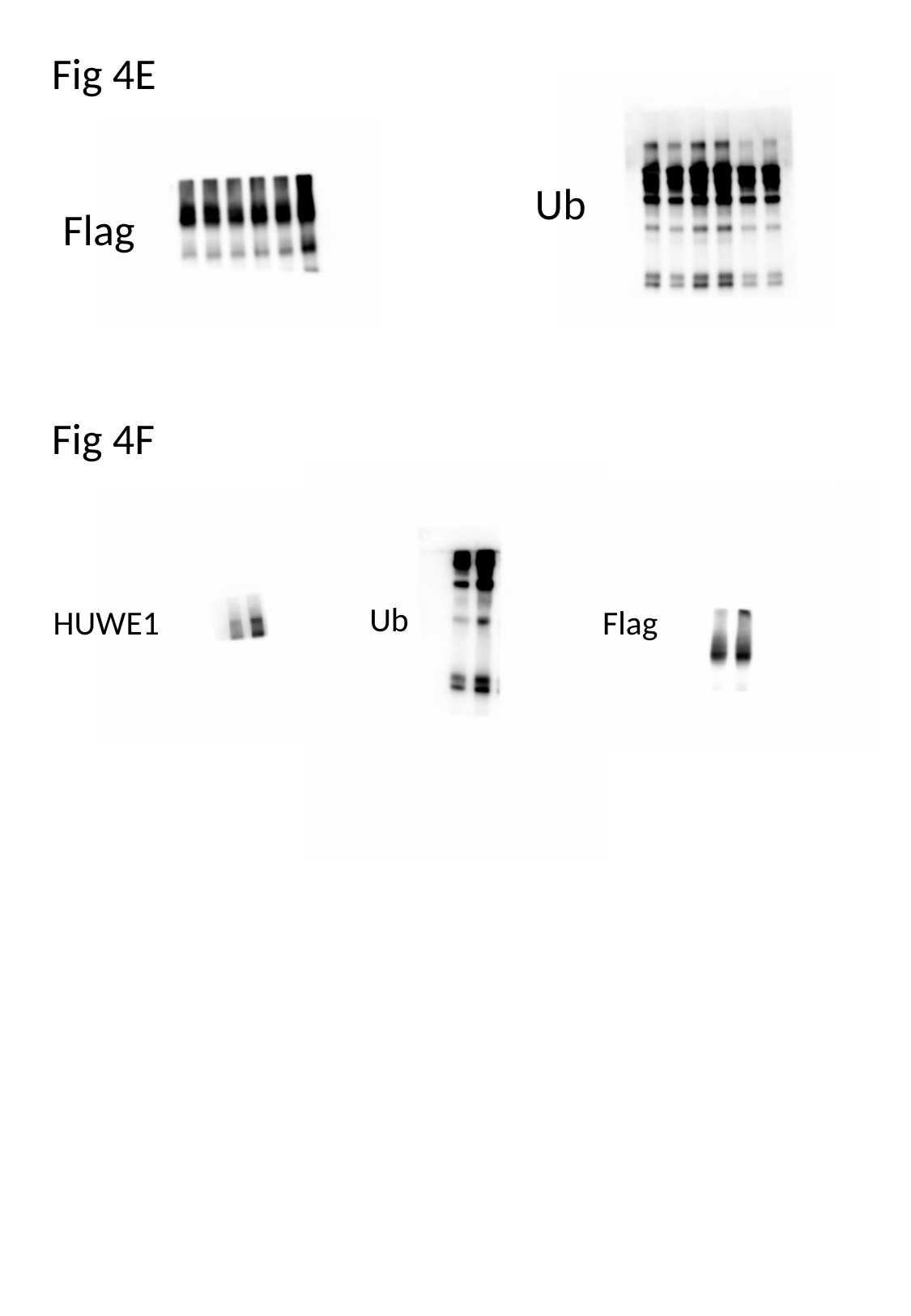

Fig 4E
Ub
Flag
Fig 4F
Ub
HUWE1
Flag

## Slide 13
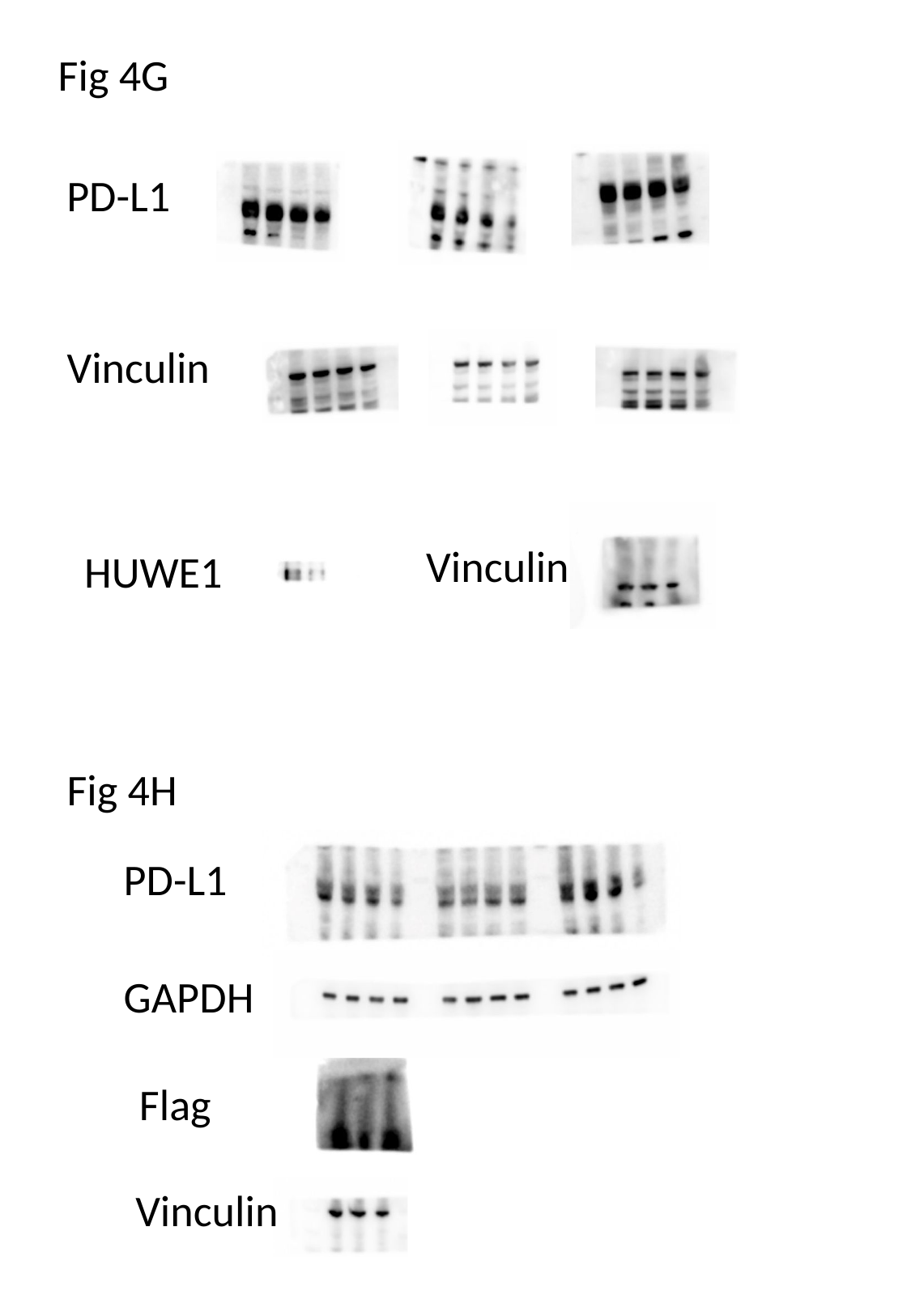

Fig 4G
PD-L1
Vinculin
Vinculin
HUWE1
Fig 4H
PD-L1
GAPDH
Flag
Vinculin

## Slide 14
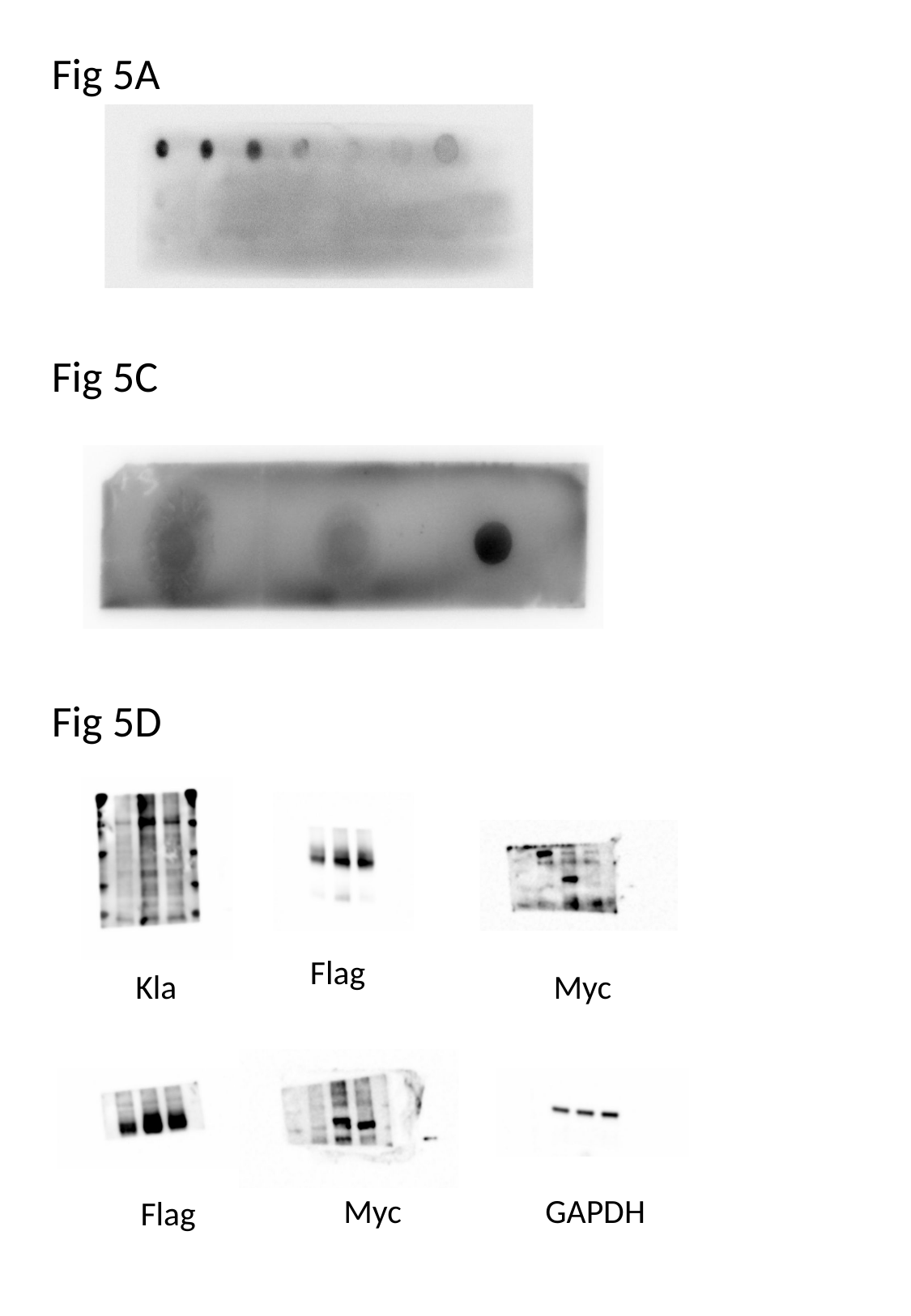

Fig 5A
Fig 5C
Fig 5D
Flag
Kla
Myc
GAPDH
Myc
Flag

## Slide 15
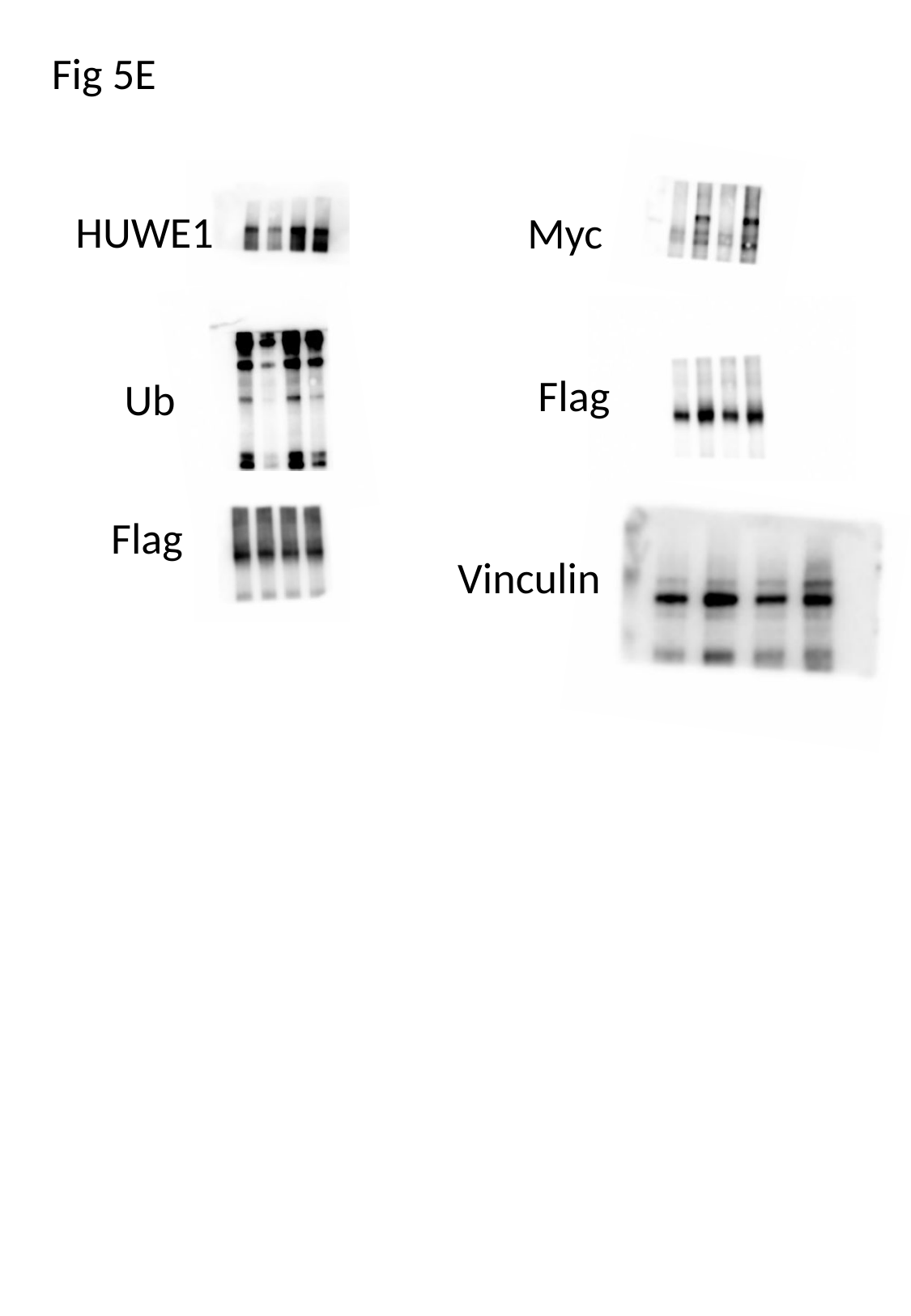

Fig 5E
HUWE1
Myc
Flag
Ub
Flag
Vinculin

## Slide 16
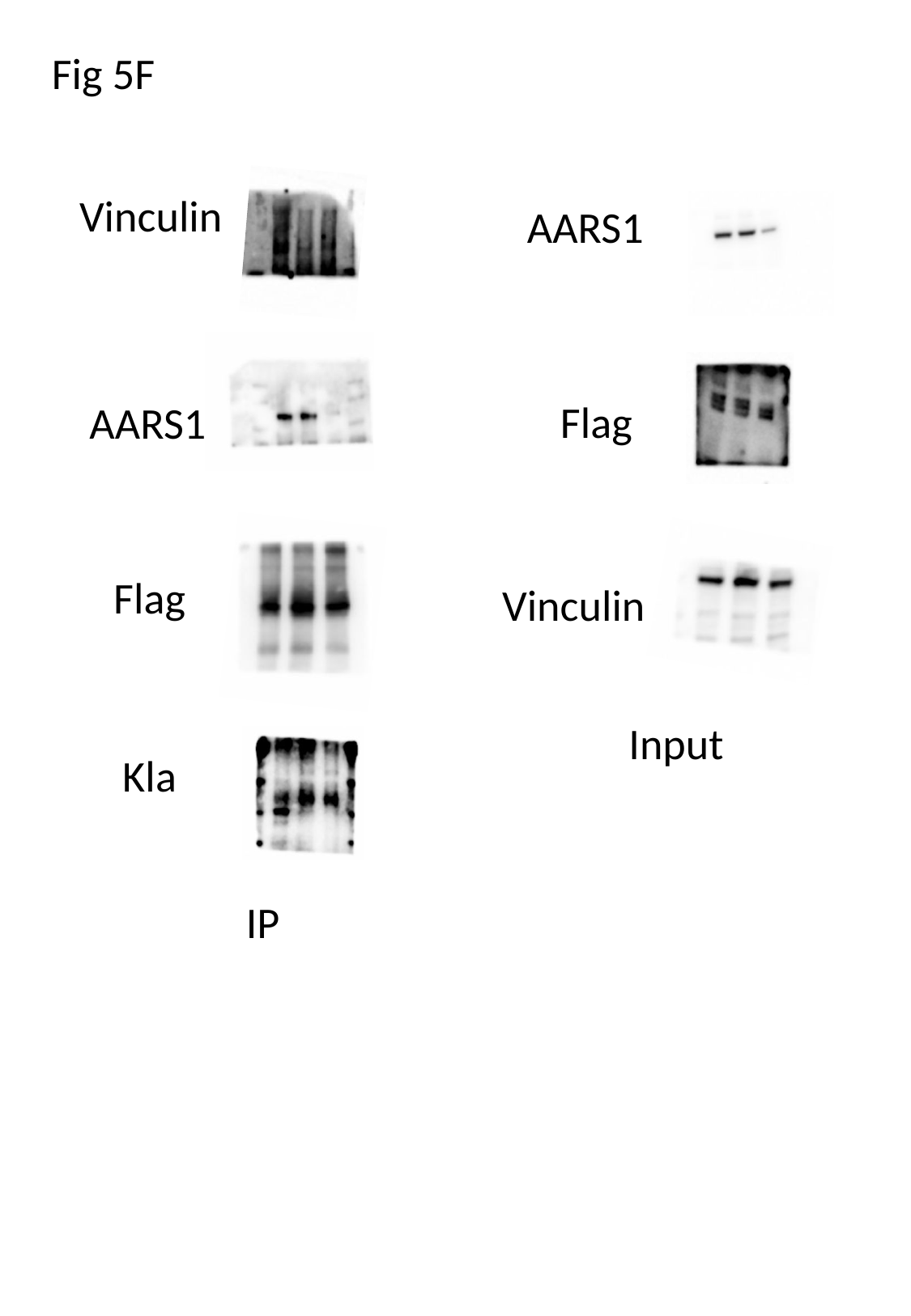

Fig 5F
Vinculin
AARS1
Flag
AARS1
Flag
Vinculin
Input
Kla
IP

## Slide 17
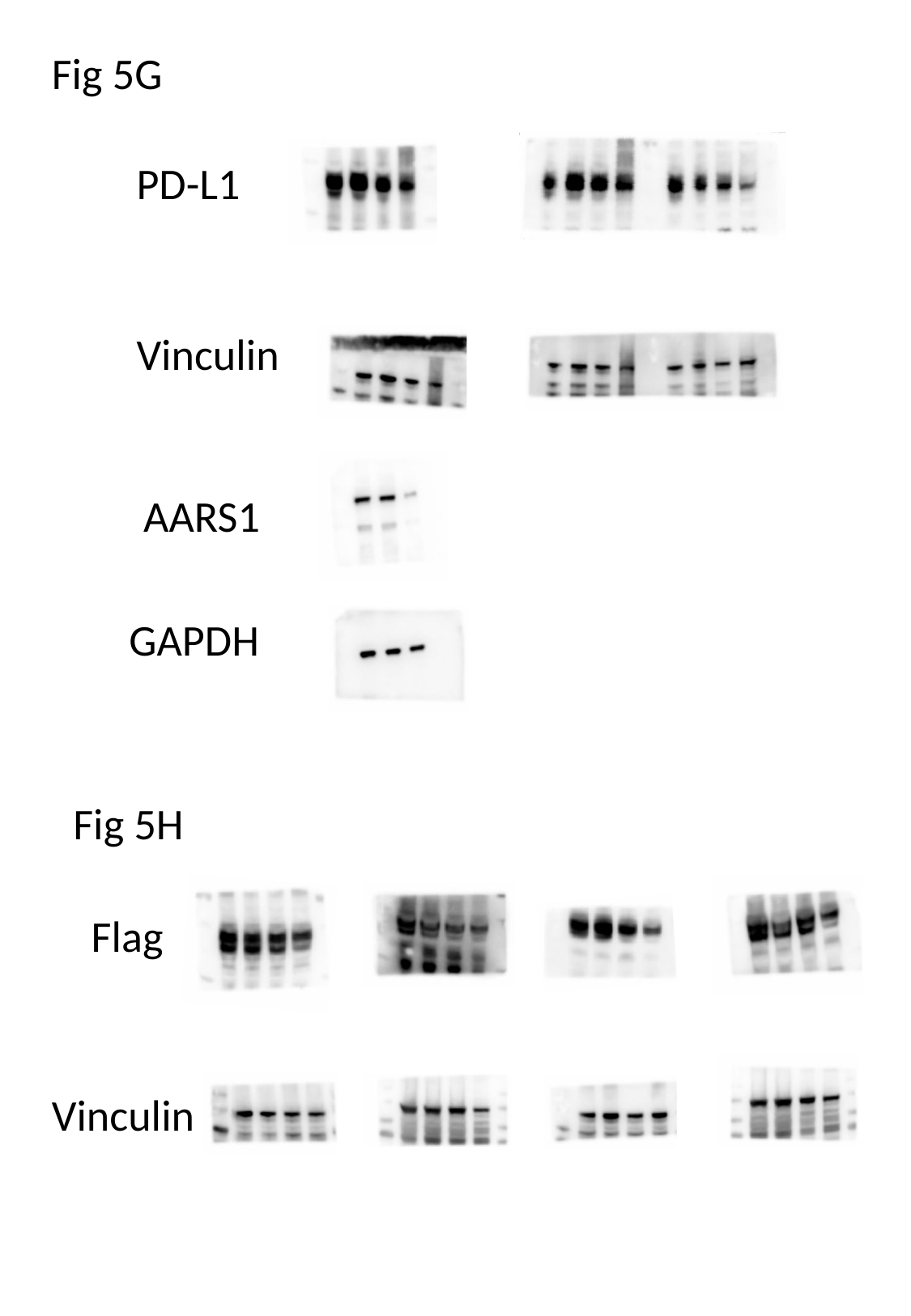

Fig 5G
PD-L1
Vinculin
AARS1
GAPDH
Fig 5H
Flag
Vinculin

## Slide 18
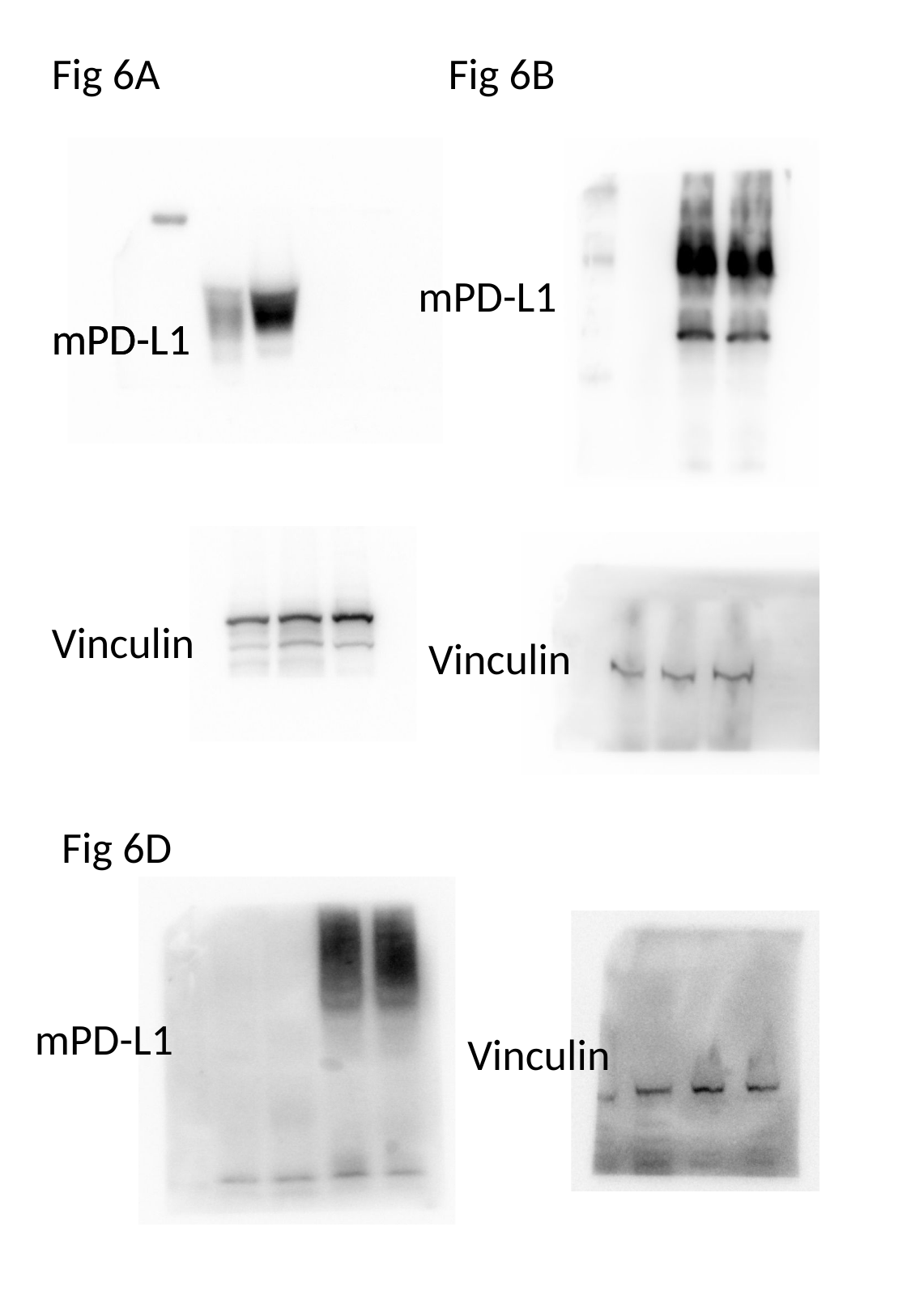

Fig 6A
Fig 6B
mPD-L1
mPD-L1
mPD-L1
Vinculin
Vinculin
Fig 6D
mPD-L1
Vinculin

## Slide 19
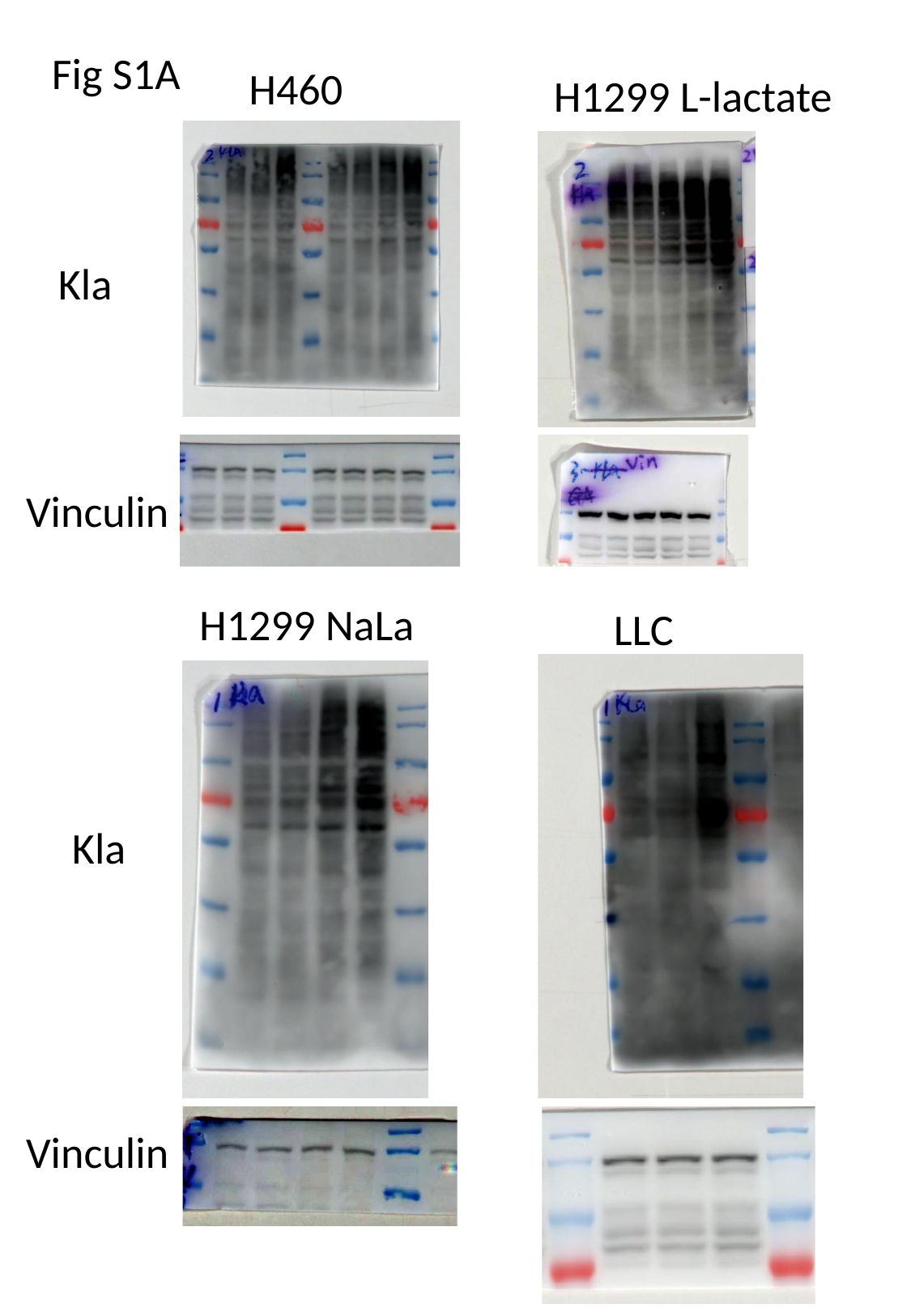

Fig S1A
H460
H1299 L-lactate
Kla
Vinculin
H1299 NaLa
LLC
Kla
Vinculin

## Slide 20
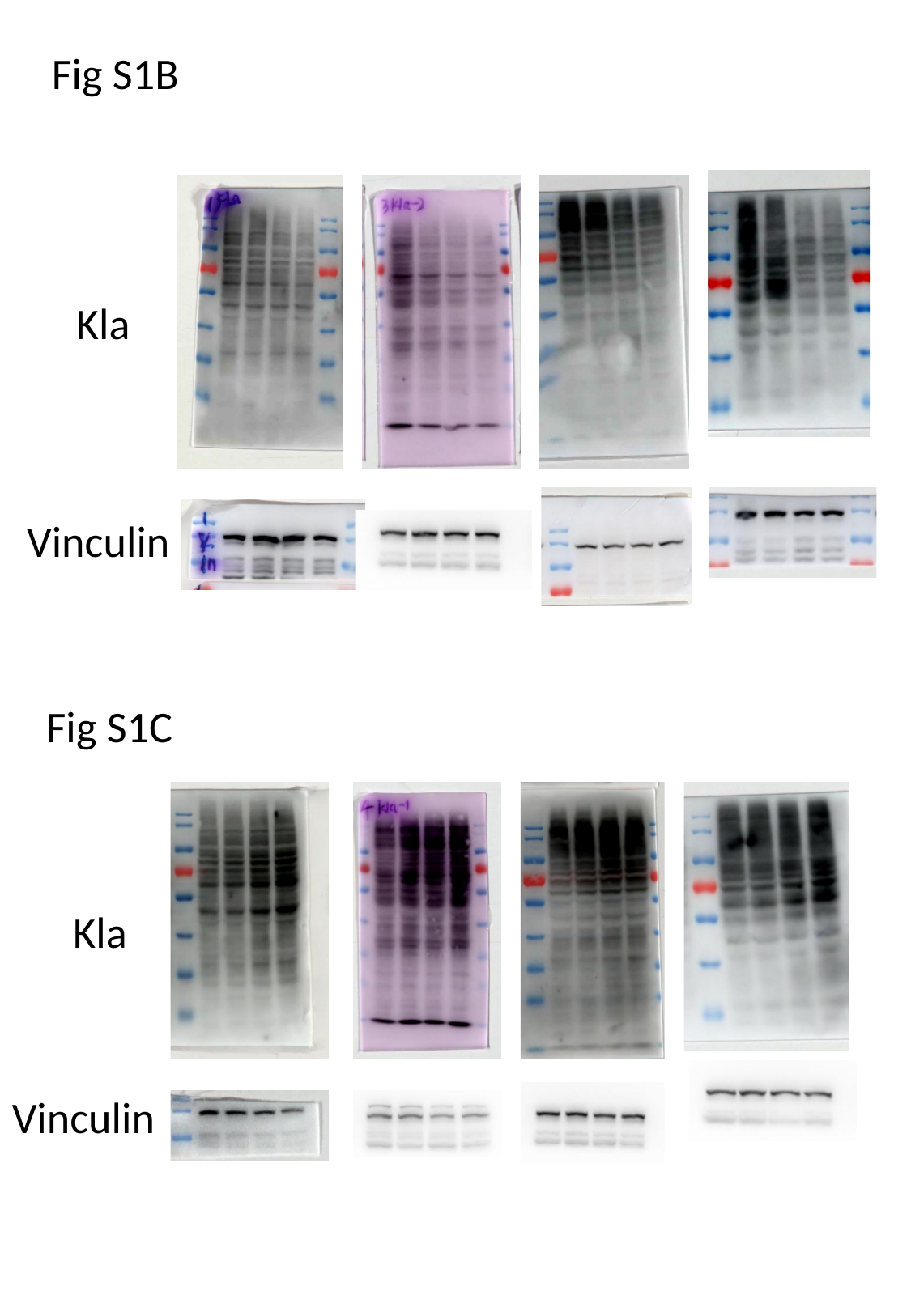

Fig S1B
Kla
Vinculin
Fig S1C
Kla
Vinculin
